# Rigid Hollow Microparticles for Enhanced Focused Ultrasound Treatment Under Optoacoustic Guidance

**DOI:** 10.1002/advs.202512337

**Published:** 2025-12-22

**Authors:** Nima Mahkam, Yi Chen, Héctor Estrada, Ananya Amitabh, Amirreza Aghakhani, Metin Sitti, Daniel Razansky

**Affiliations:** ^1^ Institute of Pharmacology and Toxicology Faculty of Medicine University of Zurich Zurich Switzerland; ^2^ Physical Intelligence Department Max Planck Institute for Intelligent Systems Stuttgart Germany; ^3^ Institute for Biomedical Engineering Department of Information Technology and Electrical Engineering ETH Zurich Zurich Switzerland; ^4^ Institute of Biomaterials and Biomolecular Systems University of Stuttgart Stuttgart Germany; ^5^ School of Medicine Koç University Istanbul Turkey; ^6^ College of Engineering Koç University Istanbul Turkey

**Keywords:** acoustophysics, focused Ultrasound, functional materials, hyperthermia, microparticles, microrobotics, optoacoustic

## Abstract

The efficacy and safety of focused ultrasound (FUS) treatments can be significantly enhanced with microbubbles, but the common ultrasound contrast agents suffer from limited stability, short circulation times, and risks associated with inertial cavitation and jetting. Here, we demonstrate that rigid hollow microparticles enable controlled, targeted thermal treatments of deep tissues via FUS. These acoustically responsive agents exhibit properties comparable to microbubbles yet possess superior mechanical stability, prolonged circulation, and enhanced responsiveness. Characterized by a negative acoustic contrast factor, the hollow microparticles amplify FUS‐induced effects—particularly localized hyperthermia—enabling precise, robust, and controllable thermal therapy. Tissue ablation experiments under optoacoustic imaging guidance demonstrate strong responsiveness to FUS, with histological analyses confirming a threefold increase in ablation volume compared with microparticle‐free controls. Experimental and numerical results indicate that this enhanced efficacy arises from first‐order acoustic effects and secondary mechanisms, including acoustic scattering and stable particle‐to‐particle interactions. Unlike microbubbles, hollow microparticles rely on non‐cavitational heating, enabling predictable, dose‐dependent thermal responses that improve safety and efficacy. The frequency‐dependent response further highlights their multifunctional potential under varying acoustic conditions. These findings establish rigid hollow microparticles as stable, versatile acoustic agents that significantly advance the therapeutic scope and clinical utility of FUS therapies.

## Graphical abstract

By combining focused ultrasound (FUS) and acoustically active hollow borosilicate microparticles (HBMPs), we demonstrate tunable control over interparticle dynamics and their localized thermal energy deposition. Modulation of acoustic frequency and particle concentration tailors the energy delivery, allowing adaptive, material‐based regulation of ultrasound‐induced effects within complex biological environments. The latter is facilitated by optoacoustic imaging feedback, thus advancing precision and efficacy in minimally invasive therapeutic applications.

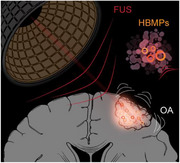

## 1 Introduction

Focused ultrasound (FUS) utilizes guided acoustic waves to precisely target specific anatomical regions, characterized by pronounced spatial gradients of sound pressure within the focal zone. Depending on the applied acoustic power, FUS is classified as low‐intensity (LIFU) or high‐intensity (HIFU), both of which have gained significant interest for their unique benefits in clinical applications [1]. LIFU is primarily used for non‐thermal applications, such as neuromodulation and transient enhancement of blood–brain barrier (BBB) permeability to facilitate drug delivery in neurodegenerative disorders, including amyotrophic lateral sclerosis (ALS) and Alzheimer's disease [2]. In contrast, HIFU is employed for thermal ablation and mechanical disruption of targeted tissues, as demonstrated in procedures like thalamotomy for Parkinson's disease [3] and treatment of malignant brain tumors, notably glioblastoma [4]. In support of these clinical applications, advancements in transcranial FUS technology have recently enabled precise, noninvasive targeting and modulation of intracranial bioeffects through the intact skull [5], markedly reducing the neurological, vascular, and postoperative complications associated with conventional surgical interventions.

HIFU has been evaluated as a non‐invasive, high‐precision therapeutic intervention to effectively destroy metastatic lesions in the brain [6]. However, HIFU ablation has typically been limited to centrally located cerebral diseases to mitigate the risk of collateral damage and overheating of healthy brain tissue and skull bone [7]. As an alternative, the use of microbubble contrast agents in conjunction with FUS has been introduced as a practical method for enhancing controlled delivery of the FUS energy to the targeted region. Bubble‐based therapeutic techniques have expanded the application possibilities of FUS by reducing the required sound intensities, thereby minimizing risks to surrounding healthy tissues [8]. This enhancement arises from the compressibility mismatch between the gaseous core of the ultrasound (US) contrast agents and the surrounding environment—blood and tissue, which increases selective acoustic absorption or facilitates destructive cavitation processes [9].

The role of microbubbles in enhancing the efficacy of HIFU therapies, particularly in thermal treatments, has been continuously reported [10]. Simulations based on experimentally derived data from large animal models have demonstrated the microbubbles’ ability to increase the treated tissue volumes near the skull base without causing significant skull heating [11], thus effectively tackling the challenges associated with HIFU therapies in the brain. Furthermore, studies using microbubbles have documented concentration‐dependent effects, resulting in nonlinear tissue damage and, hence, diminished collateral damage to surrounding healthy tissues compared to control (microbubble‐free) conditions [12]. The relationship between microbubble concentration and the safety profile of FUS‐mediated applications has been explored across a range of FUS parameters and injection dosages to maximize the therapeutic index while minimizing potential adverse effects [13, 14].

Advances in image‐guided therapeutic methods [15] have enabled real‐time monitoring and adjustment of FUS effects. A range of different imaging modalities have been used for these purposes, including magnetic resonance imaging (MRI) [15, 16], ultrafast ultrasound [17], and optoacoustic (OA) imaging – all capable of volumetric imaging with adaptable acquisition capabilities [18]. However, despite the integration of real‐time monitoring during FUS procedures, current clinical FUS thermo‐ablation methods continue to pose significant risks of injuries to bone marrow [19, 20], vascular, and parenchymal tissues [21]. Cavitation‐based approaches elevate the risk of injury to sensitive neurovascular structures due to the inherently abrupt and destructive nature of cavitation [22], whilst other shortcomings include the instability and short circulation lifetime of bubble‐based agents [23, 24]. Therapeutic variability associated with microbubbles highlights the need for alternative modalities that provide greater stability, controllability, and enhanced acoustic responsiveness compared with bubble‐based agents. Noncavitational methods offer additional advantages, including improved precision, reduced surgical and post‐surgical risks, and a higher therapeutic index that is less dependent on local tissue properties. Furthermore, concerns regarding nanoparticle toxicity [25] and the limited ability of systemically administered agents to cross the BBB and penetrate the tumor core [26, 27] underscore the value of strategies that leverage the long‐term stability of particles delivered intratumorally. The intratumoral administration route addresses these limitations by achieving higher, consistent local particle concentrations, thereby reducing the risks of embolism and toxicity associated with systemic exposure. This approach confines the therapeutic effect to the particle‐exposed region and the FUS focal volume, enabling repeated treatments over extended periods and reducing invasiveness.

Here, we introduce a new mechanism to further enhance FUS therapeutic efficacy using rigid, hollow, biocompatible microparticles. The microparticles consist of thin borosilicate shells with an air‐filled core, which effectively mitigate cavitation‐associated risks while maintaining a negative acoustic contrast factor (ACF) and significantly prolonged circulation lifetimes. We characterized their hyperthermic response and demonstrated their efficacy in tissue ablation with FUS. Additionally, we report frequency‐ and dose‐dependent responses analogous to microbubble behavior, monitored in real time and in 3D using OA tomography. Our findings elucidate that the thermal dose‐dependent responses of microparticles are driven by acoustic scattering and secondary Bjerknes forces (particle‐particle interactions), functioning as either heat dissipators or acoustic wave reflectors. The observed frequency‐dependent response enables multifunctionality in particle‐based approaches, including acoustic trapping under high flow conditions and precise thermal modulation. These results highlight the potential for transitioning conventional microbubble‐based FUS enhancement approaches to hollow microparticle‐based strategies, offering superior control, stability, and predictability in therapeutic applications.

## 2 Results

### 2.1 Simultaneous Acoustic Manipulation and Optoacoustic Imaging of Hollow Microparticles

**FIGURE 1:**
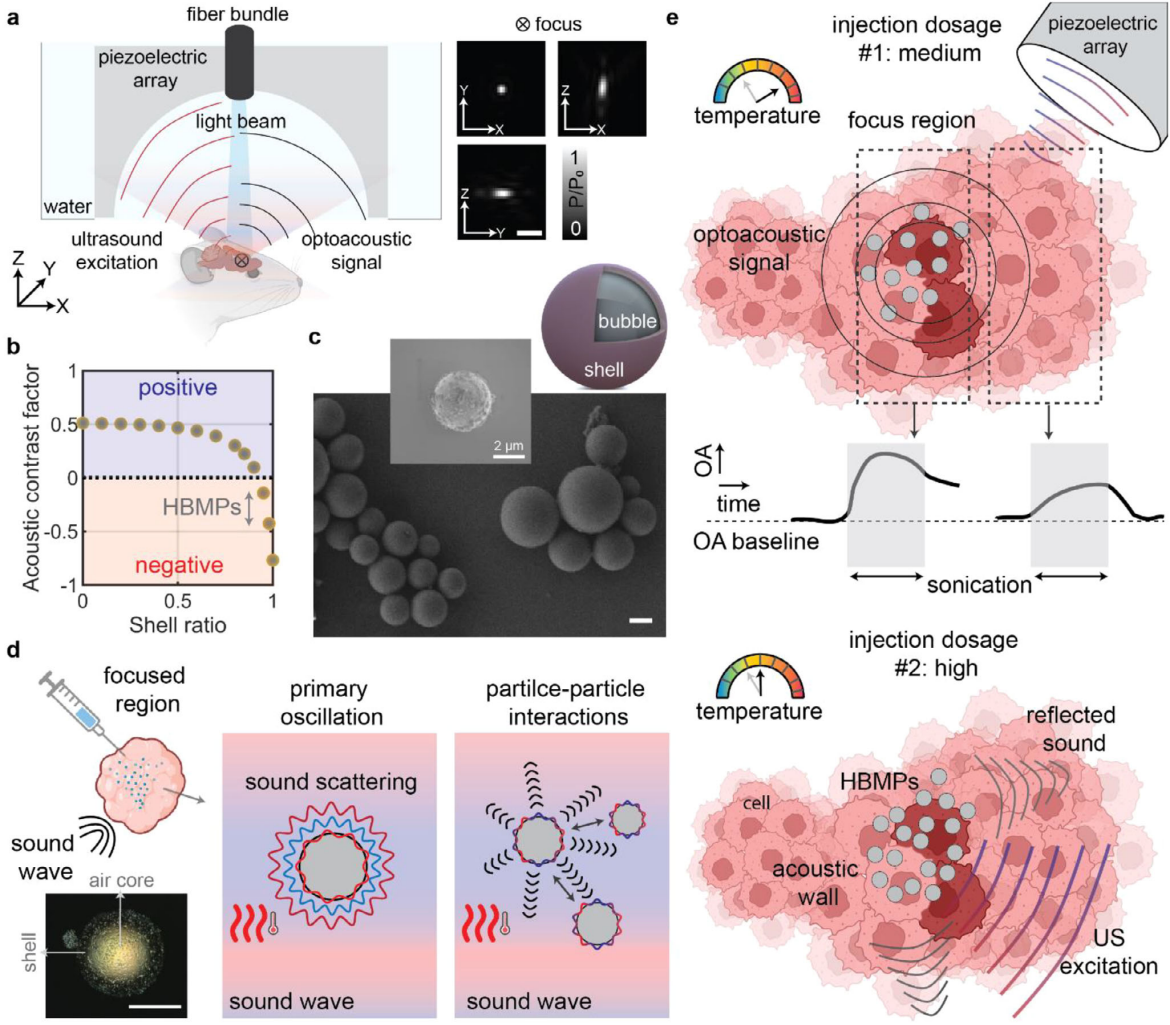
Integrated optoacoustic (OA) imaging and focused ultrasound (FUS) manipulation system, and characterization of hollow borosilicate microparticles (HBMPs). (a) System schematic. Normalized acoustic pressure maps are shown across three orthogonal planes. Scale bar: 0.5 mm. (b) Acoustic contrast factor of hollow microparticles and its correlation with shell thickness. (c) Scanning electron microscopy images of HBMPs in bulk and as individual particles. Scale bar: 2 µm. The microparticles feature a gaseous (air) core encapsulated by a thin borosilicate shell. (d) The acoustic behavior of gas‐core HBMPs governed by both primary and secondary acoustic radiation forces. Primary forces induce scattering, while secondary forces drive inter‐particle interactions, contributing to observed thermal effects. Confocal laser microscopy of a single HBMP, with the air core and shell. Scale bar: 10 µm. (e) Dose‐dependent thermal responses of HBMPs detected by OA imaging, demonstrating a dual role as either thermal agents or acoustic walls/reflectors that modulate local tissue temperature fluctuations.

The simultaneous FUS manipulation and OA imaging setup consists of a wide‐angle spherical ultrasound array formed by 512 piezoelectric elements, with an optical fiber centrally positioned to deliver excitation light pulses (see Materials and Methods for details). The piezoelectric elements can flexibly switch between receiving and emitting phases [28], capturing dynamic changes of the sample induced by particles under FUS and accurately steering the focal point (focus) to target microparticles (Figure 1a). The FUS beam generated by the array at 5 MHz exhibits an elongated elliptical shape, with a volume of 0.05 mm^3^, 200 µm lateral dimension, and 400 µm axial dimension as measured by its full width at half maximum (Figure 1a).

The hollow borosilicate microparticles (HBMPs) used in this study consist of a thin shell encapsulating an air bubble core. Their acoustic properties enable behavior similar to that of microbubbles, but with reduced wall‐oscillation amplitudes and enhanced stability, attributed to the approximately 200 nm‐thick borosilicate shell (Figure S1) [29]. Additionally, the shell thickness plays a critical role in defining the microparticle's compressibility, which directly influences the range of the ACF [30, 31]. Theoretical analysis correlated the shell thickness with the achieved ACF for hollow microparticles, while experimental measurements determined an average ACF of −0.25 for HBMPs (Figure 1b; Figures S2–S4; Sections S1 and S2).

HBMPs have a mean diameter of 12 µm before filtration (Figure 1c) and 5 µm after filtration (see Section S3, and Figure S5 for further details). The 200‐nm‐thick borosilicate shell of the HBMPS provides superior mechanical stability and structural integrity under clinically relevant pressure conditions (see Figure S6 and Section S4). The shell effectively mitigates deformation and collapse during sonication, preserving the gaseous core, while maintaining the intrinsic ACF, ensuring consistent acoustic performance in therapeutic settings. The ACF and density range of HBMPS allow them to emulate the behavior of microbubbles in FUS therapies, while several physical principles governing microbubble dynamics can, to a certain extent, be extended to microparticles. This includes the thermal response and its correlation with particle‐to‐particle interactions driven by the secondary Bjerknes force [32, 33]. The microbubble interaction with US has been reported to involve two fundamental aspects, namely, the primary and secondary interactions [34, 35]. The primary interaction—primary acoustic forces—involves particles oscillating solely due to propagating sound waves, followed by acoustic scattering. The interaction of these scattered waves with other particles gives rise to secondary forces (Figure 1d). While the thermal response of microbubbles has primarily been associated with cavitation effects, studies have shown that microbubble oscillation amplitudes influence temperature increases, amplifying thermal effects at the focal point and thereby improving the efficiency of HIFU therapies [36]. This behavior is hypothesized to apply to HBMPs, in which both scattered sound waves and secondary Bjerknes forces contribute to localized heating and a temperature increase during FUS sonication (Figure 1d).

Size inherently influences the primary acoustic effects, through FUS‐particle interactions, which directly affect the resulting heating performance (Figure S7 and Section S5); however, heterogeneity in bubble size has also been shown to enhance secondary acoustic interactions [37]. Particularly, studies have shown that smaller bubbles tend to experience substantial suppression effects due to bubble‐bubble interactions and secondary Bjerknes forces [38], whereas larger bubbles can exert greater forces owing to their increased surface area and volume [38, 39]. The size heterogeneity of HBMPs in this study critically influences the thermal dynamics observed, similar to microbubbles. The interactions between smaller and larger particles amplify scattering and secondary forces, facilitating enhanced heat dissipation. Another acoustic phenomenon transferable from microbubbles to microparticles is their concentration‐dependent response [40]. Our experiments demonstrate that for a given frequency, a specific particle concentration range enhances thermal response while exceeding a certain threshold leads to diminished heating effects, regardless of the FUS frequency (Figure 1e).

### 2.2 Characterization of Frequency‐ and Concentration‐Dependent Acoustic Response of HBMPs

**FIGURE 2:**
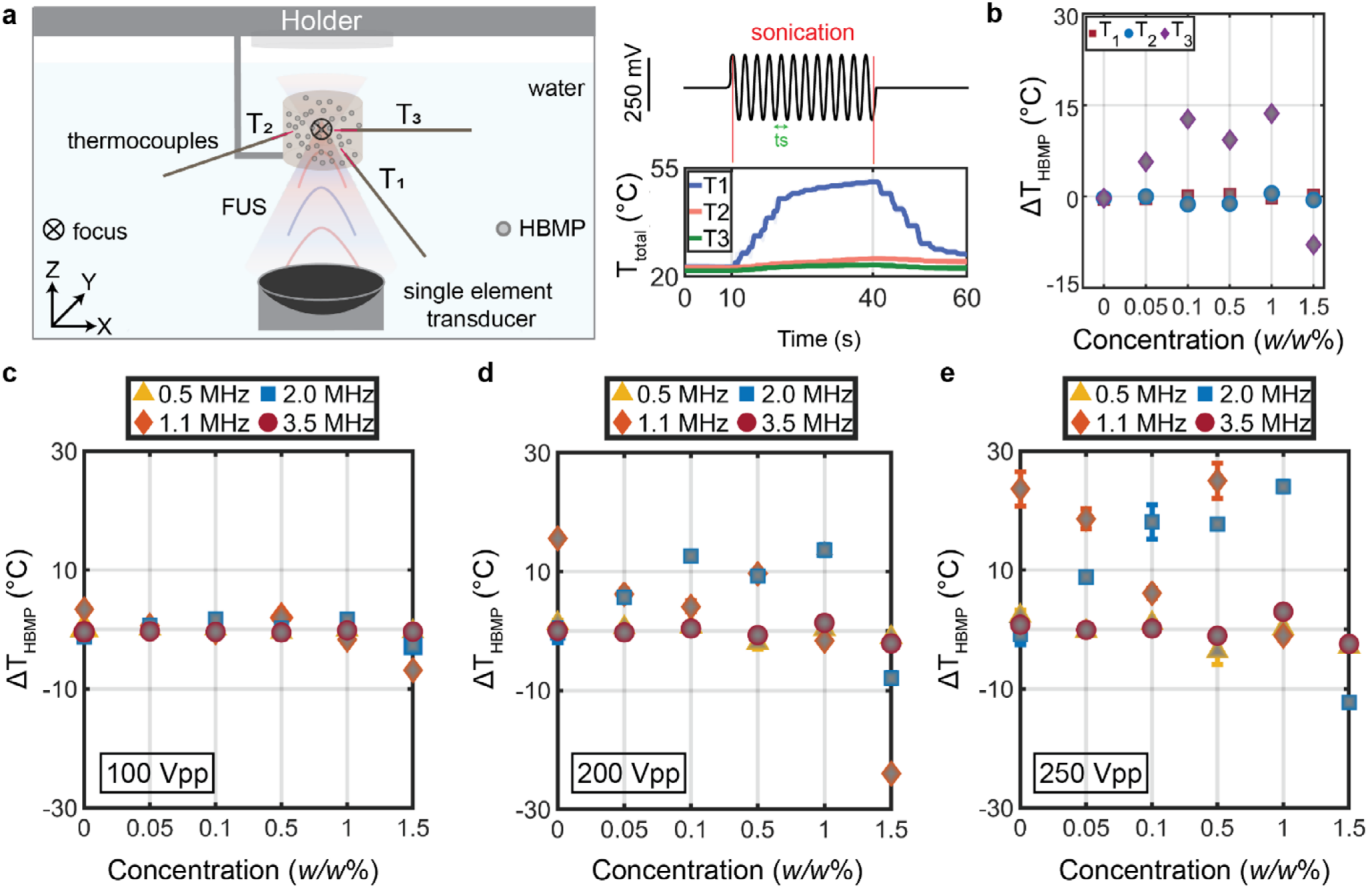
Concentration‐ and frequency‐dependent heating response of HBMPs. (a) The experimental setup with single‐element transducers and thermocouple temperature recordings over time. (b) Relative temperature measurements from three thermocouples at 2 MHz FUS exposure across varying HBMP concentrations. (c–e) Maximum temperature recorded after 30 s of FUS sonication at an input voltage of 100 (c), 200 (d), and 250 mVpp (e). All results represent mean values of three independent trials, with error bars indicating standard deviation.

To elucidate the HBMPs interaction with FUS and their corresponding heating response, we used single‐element transducers, each characterized by a different resonance frequency of 0.5, 1.1, 2, and 3.5 MHz, an optical thermocouple with three channels, a water tank, and a polydimethylsiloxane (PDMS) chamber (Figure 2a; Figure S8).

The heating response of HBMPs has been assessed under various sound pressure fields and concentrations. To isolate the thermal contribution of HBMPs, we normalized the temperature readings (Δ*T_total_*) with the FUS‐induced heating of pure water without particles (Δ*T_FUS_*) under identical exposure durations and acoustic parameters (Figure S9), measured prior to each test as Δ *T_HBMP_* =  Δ*T_total_* − Δ*T_FUS_* (see Materials and Methods). Positive Δ*T_HBMP_* corresponds to a higher temperature rise with respect to the water baseline, while negative Δ*T_HBMP_* indicates reduced heating. Comparing the maximum Δ*T_HBMP_* measured at different locations relative to the FUS focus (Figure 2a), we clearly observe a consistent variation only for the thermocouple located closer to the focus region (Figure 2b).

In this study, six distinct weight‐to‐weight (*w/w*) mixing ratios were used to evaluate the concentration‐ and frequency‐dependent behavior of scattered heat and temperature. Although a direct comparison between different FUS frequencies is not feasible due to inherent variations in focal size governed by the acoustic wavelength, the temperature readings indicate that each frequency produces an optimal temperature increase at specific particle concentrations (Figure 2c–e). For instance, at 2 MHz, particle concentrations between 0.1% and 1% induce a temperature increase of approximately 20°C (Figure 2e), which is particularly relevant for hyperthermic applications [41].

Independent of frequency, increasing the mixing ratio beyond a certain threshold tends to negatively affect the heating properties. For example, a Δ*T_HBMP_* of −10 at 2 MHz and a 250 mVpp voltage indicates that the medium is heated by 10 degrees less than the water baseline. This phenomenon can be attributed to the scattering of FUS waves by particles (US shadowing), which interferes with the formation of a high‐intensity focal region (acting as acoustic reflectors, Figure 1e). Consequently, particles located farther from the transducer remain unexposed, resulting in a reduced heating response.

The temperature response characterizations also indicate the role of pressure on the temperature variations (Figure 2c–e). Intuitively, higher voltage amplitudes generate greater acoustic pressure on the particles and within the focal region, with temperature differences between the particle suspensions and the baseline becoming more pronounced at higher pressures (Figure 2e) and remaining negligible at lower pressures (Figure 2c). This phenomenon can be correlated with particle‐to‐particle interactions becoming more significant at higher acoustic intensities [42]. Moreover, increasing acoustic pressure amplifies concentration‐dependent effects; at 1.1 MHz, the 0% concentration shows greater heating efficiency at 100 mVpp and 200 mVpp; however, at higher acoustic pressures, the 0.5% particle suspension achieves efficacy comparable to that of the 0% condition (Figure 2c–e). At 0.5 and 3.5 MHz, temperature variations remain close to the baseline water temperature, indicating that particle oscillations do not contribute to heating and suggesting the existence of natural resonance behavior in particle‐FUS interactions.

### 2.3 Numerical Simulations of the Thermal Response of HBMPs

**FIGURE 3:**
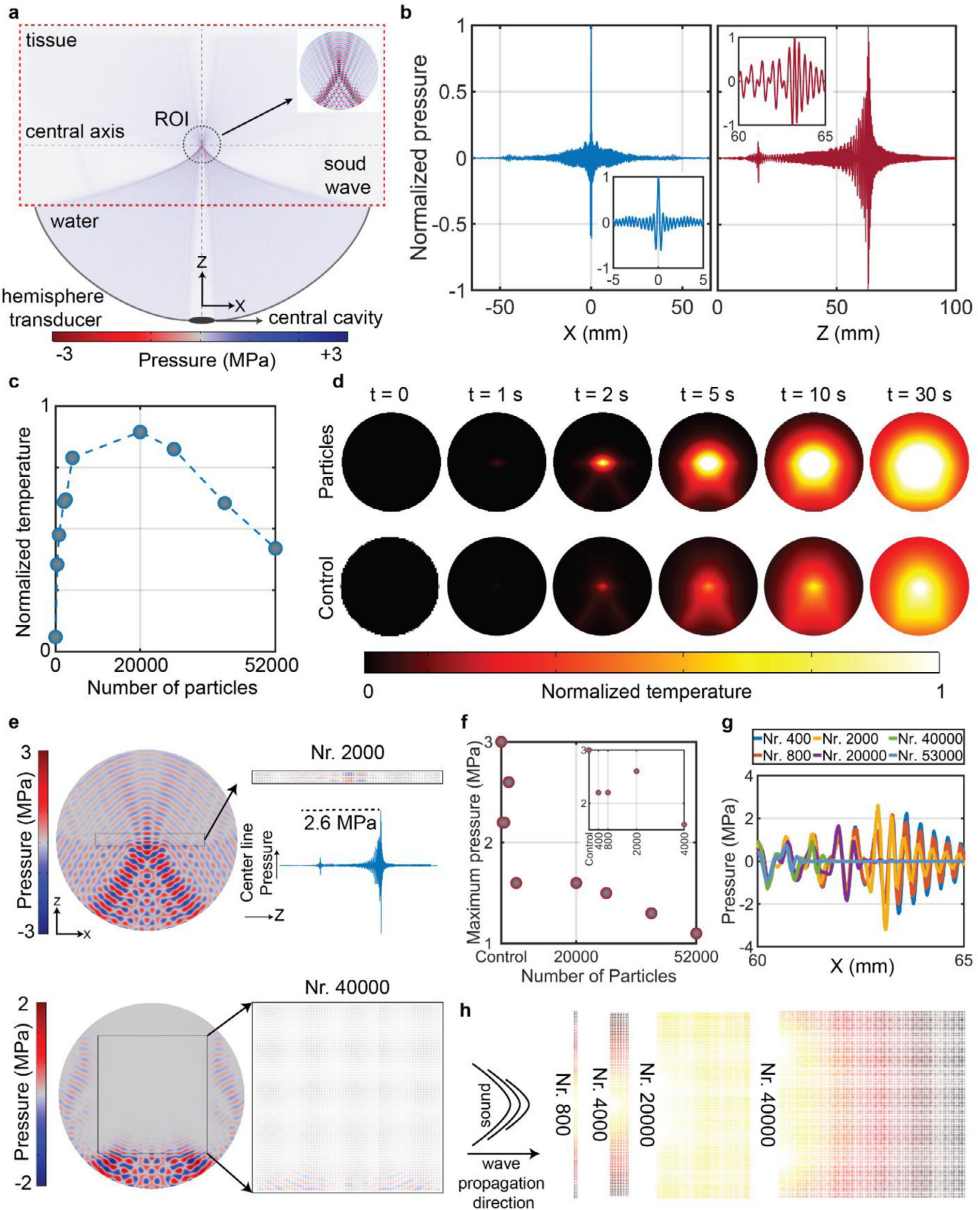
Numerical analysis of the HBMP heating performance. (a) Schematic of the simulated geometry with a hemispherical transducer filled with water and a tissue sample. A region of interest containing the particles is defined for analytical temperature estimation. (b) Simulated sound pressure distribution along two primary axes. (c) Maximum average temperature observed in the ROI after 30 s of sonication, as a function of particle count. (d) Temporal temperature profiles within the ROI for the case with HBMPs (*Nr* = 20,000), compared to the control condition with FUS‐only exposure (no particles). (e) Pressure distribution profiles in the ROI for *N_r_* = 2,000 and *N_r_* = 40,000. (f) Maximum pressure along the central axis relative to particle count. (g) Pressure profiles along the x‐axis of the ROI at varying particle concentrations. (h) Temperature distribution in the ROI as a function of *N_r_*.

To gain further insights into the impact of concentration on the thermal response of HBMPs under FUS sonication, we employed a numerical model replicating the experimental conditions (see Materials and Methods). It incorporates a hemispherical transducer filled with water and positioned adjacent to a tissue sample. The model includes a designated region of interest (ROI) containing microparticles (Figure 3a). The pressure distribution indicates a centrally located, high‐intensity acoustic field at 5 MHz, coinciding with the particles’ location (Figure 3a,b). To simulate the effect of varying concentrations, we introduced *N_r_*, a parameter representing the number of particles for a given constant ROI volume. Subsequently, the temperature fluctuations within the ROI were computationally modeled, and the results were spatially averaged 30 s post sonication. The simulated temperature response aligns with experimental observations, demonstrating that increasing the *N_r_* beyond a critical threshold negatively impacts heat deposition (Figures 2c–e and 3c). Time‐lapse images of the ROI under control conditions (FUS‐only) and with *Nr* = 20,000 microparticles are shown in Figure 3d. Comparing the control condition and the particle‐containing ROI indicates that particle presence enhances temperature elevation during sonication and facilitates heat convection across a larger volume (Figure 3d). Additionally, the bullet‐shaped temperature distribution observed in the ROI results from the intrinsic characteristics of FUS. The pressure gradually increases, reaching a high‐intensity focal region, and then sharply declines (Figure 3b), producing the observed bullet‐shaped temperature profile.

To elucidate this *N_r_*‐dependent thermal response, we investigated the influence of particles on the pressure distribution within the ROI for *N_r_* = 2,000 and *N_r_* = 40,000. In the former case, all particles are exposed to the US, whereas in the latter, only particles in the initial layers experience the US pressure (Figure 3e). Increasing the particle count beyond a critical threshold, where the dimensions of the particle‐laden region exceed the focal volume, significantly disrupts the formation of the high‐intensity region (Figure 3e). Specifically, particles located closer to the transducer act as a scattering barrier (US shadowing), which explains the experimentally observed negative effects, wherein excessive particle concentrations lead to reduced heating compared to baseline conditions (Figure 2c–e).

Additionally, we calculated the average pressure within the ROI along its central axis (Figure 3a, dotted lines). A noticeable reduction in maximum pressure intensity occurs as the *N_r_* increases due to microparticle‐induced scattering (Figure 3f). Although the ROI containing *N_r_* = 20,000 particles experiences relatively low maximum pressure compared to conditions with fewer or no particles (Figure 3f), the achieved temperature elevation is significantly higher (Figure 3c). This enhancement can be attributed to viscous dissipation and secondary acoustic effects arising predominantly from microparticle interactions under US exposure, which amplify acoustic scattering, consequently enhancing heat deposition, like microbubbles [43].

Pressure distribution along the axial dimension indicates that increasing *N_r_* progressively shifts the position of maximum US pressure closer to the transducer (Figure 3g). Consequently, particles that initially encounter the US wave are subjected to higher pressure and increased scattering effects, leading to a shift of the focal region toward the transducer. This phenomenon directly influences heat generation and temperature elevation within the particle‐containing region, as the particle layers closer to the transducer predominantly contribute to heating, while temperature elevation gradually decreases with increasing distance (Figure 3h).

### 2.4 Ex Vivo Thermal Characterization

**FIGURE 4:**
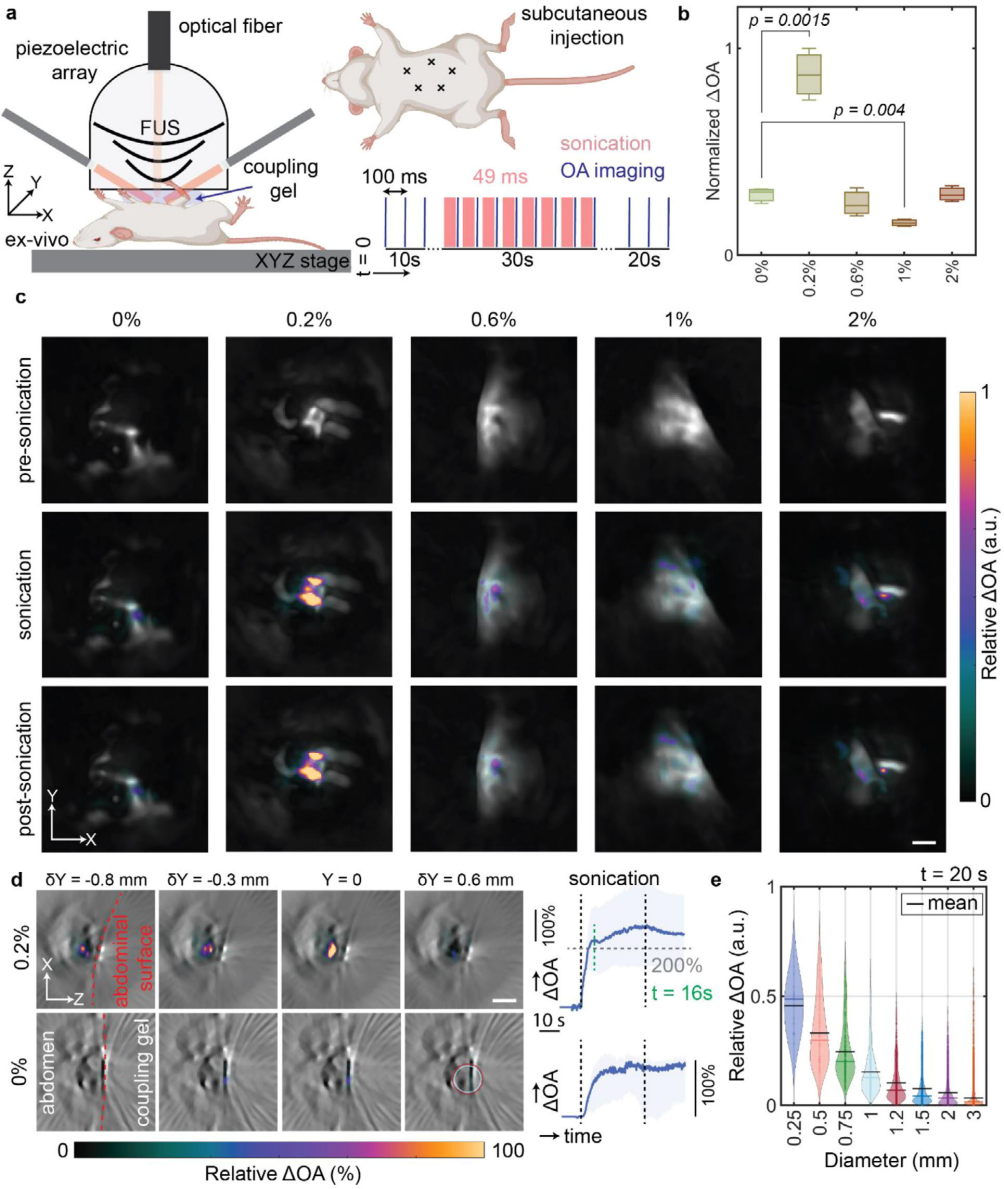
Ex vivo concentration‐dependent characterization of HBMPs. (a) The experimental schematics, including the OA‐FUS setup and the sample holder. The FUS sequence includes 10 s of pre‐sonication, 30 s of sonication, and 20 s of post‐sonication recordings. A 10 ns laser pulse is triggered via an optical fiber, and the FUS‐OA sequence is synchronized in 100 ms intervals with a 49% FUS duty cycle. (b) Relative OA signal change for varying particle concentrations, averaged over a 1 mm^2^ ROI. Statistical comparisons were performed using a two‐sided one‐way ANOVA comparing each concentration group with the 0% reference group (n = 4 per group). Significant differences were observed between the 0% and 0.2% groups (*p* = 0.0015) and between the 0% and 0.6% groups (*p* = 0.004); all other group comparisons were not statistically significant (*p* > 0.05). (c) Spatial‐ and temporal‐MIPs across three experimental phases: pre‐sonication (0–10 s), sonication (10–40 s), and post‐sonication (40–60 s). Scale bar: 1 mm. (d) Sliced temporal‐ and spatial‐maximum intensity projection (MIP) images showing the relative OA signal changes for 0.2% and 0% in the xz‐plane, illustrating volumetric changes and activation depth. MIPs are captured 10 seconds after sonication onset and span 0.1 mm above and below the selected plane. The time‐lapse ΔOA plots show relative signal changes averaged over a 0.25 mm diameter ROI. Scale bar: 1 mm. The shaded area represents the distribution range, showing the minimum and maximum values. (e) Relative OA signal changes for 0.2% particle concentration, plotted as a function of ROI diameter, measured 10 seconds after the onset of sonication. The results show the mean and distribution of four different samples.

For the subsequent experiments, we identified the particle concentration compatible with the 5 MHz operating frequency of our integrated FUS‐OA device. The experimental setup (Figure 4a) consists of a piezoelectric transducer array (Figure 1a) and a manual positioning stage for sample alignment. The combined FUS and imaging protocol comprises 100 ms intervals between each laser pulse, with 49 ms dedicated to sonication (Figure 4a). Experiments were conducted with an initial 10‐s pre‐sonication recording period (OA_0_) to establish a baseline for the relative OA signal change, defined as ΔOA_n_ = (OA‐OA_0_)/OA_0_. This was followed by 30 s of sonication and an additional 20 s of post‐sonication monitoring (Figure 4a). Mixtures with varying concentrations of HBMPs suspended in phosphate‐buffered saline (PBS) were administered (20 µL each) into the mouse abdominal region. Five sonication points were identified that aligned with the injection sites (Figure 4a). The observed maximum relative OA signal changes within a 1 mm^3^ ROI across different experimental conditions exhibit a particle concentration‐dependent pattern (Figure 4b). Specifically, the 0.2% concentration demonstrates a greater OA signal change under similar FUS parameters.

Another notable difference between varying concentrations relates to the extent of the affected region. Using the optimal concentration at the given frequency increases the affected volume, as observed in the distribution of the maximum intensity projections (MIPs) of the ΔOAn at three time periods—pre‐sonication (0–10 s), during sonication (10–40 s), and post‐sonication (40–60 s) (Figure 4c). Temporal‐MIPs of ΔOA_n_ over 10 s following the initiation of FUS under 0% and 0.2% conditions reveal enhanced penetration and an expanded affected area in the lateral plane (Figure 4d).

Additionally, 10 s after sonication, the HBMPS solution exhibits a signal change approximately twofold greater than that observed in the particle‐free condition (Figure 4d). The increasing trend in the OA signal (Figure 4d, ΔOA time‐lapse graphs) correlates with rising temperature, and a sudden change in the OA signal pattern indicates an onset of tissue coagulation [44]. The OA signal trend changes after ∼6 s of sonication (*t* = 16s), potentially indicating changes in tissue optical properties. Enlarging the ROI diminishes the observed changes, with averaged ΔOA_n_ approaching zero at distances exceeding 1 mm from the focus (Figure 4e). This observation suggests an affected area of approximately 0.75 mm (half‐maximum) at a 0.2% particle concentration ratio and 5 MHz, coupled with a sonication period of 30 s (Figure 4e).

The frequency‐ and concentration‐dependent analysis previously verified in phantoms (Figure 2c–e) and ex vivo samples (Figure 4b)—demonstrating distinct active and inactive states based on the applied FUS frequency—underscores the potential for multifunctional capabilities in particle‐based therapeutic strategies. This approach enables precise, on‐demand activation for controlled thermal responses, as well as additional functionalities such as acoustic trapping (Figure S10). The trapping efficacy of HBMPs under continuous‐flow conditions at 3 MHz was evaluated using a setup analogous to the integrated FUS‐OA system, employing a FUS waveform optimized for particle trapping. Effective trapping at flow velocities up to 25 mm/s (Figure S10) supports the feasibility of multifunctional particle‐based platforms capable of localized manipulation following intravenous administration, combined with frequency‐selective thermal activation (at 5 MHz) for thermal therapeutic application.

### 2.5 Brain Hyperthermia and Histology

**FIGURE 5:**
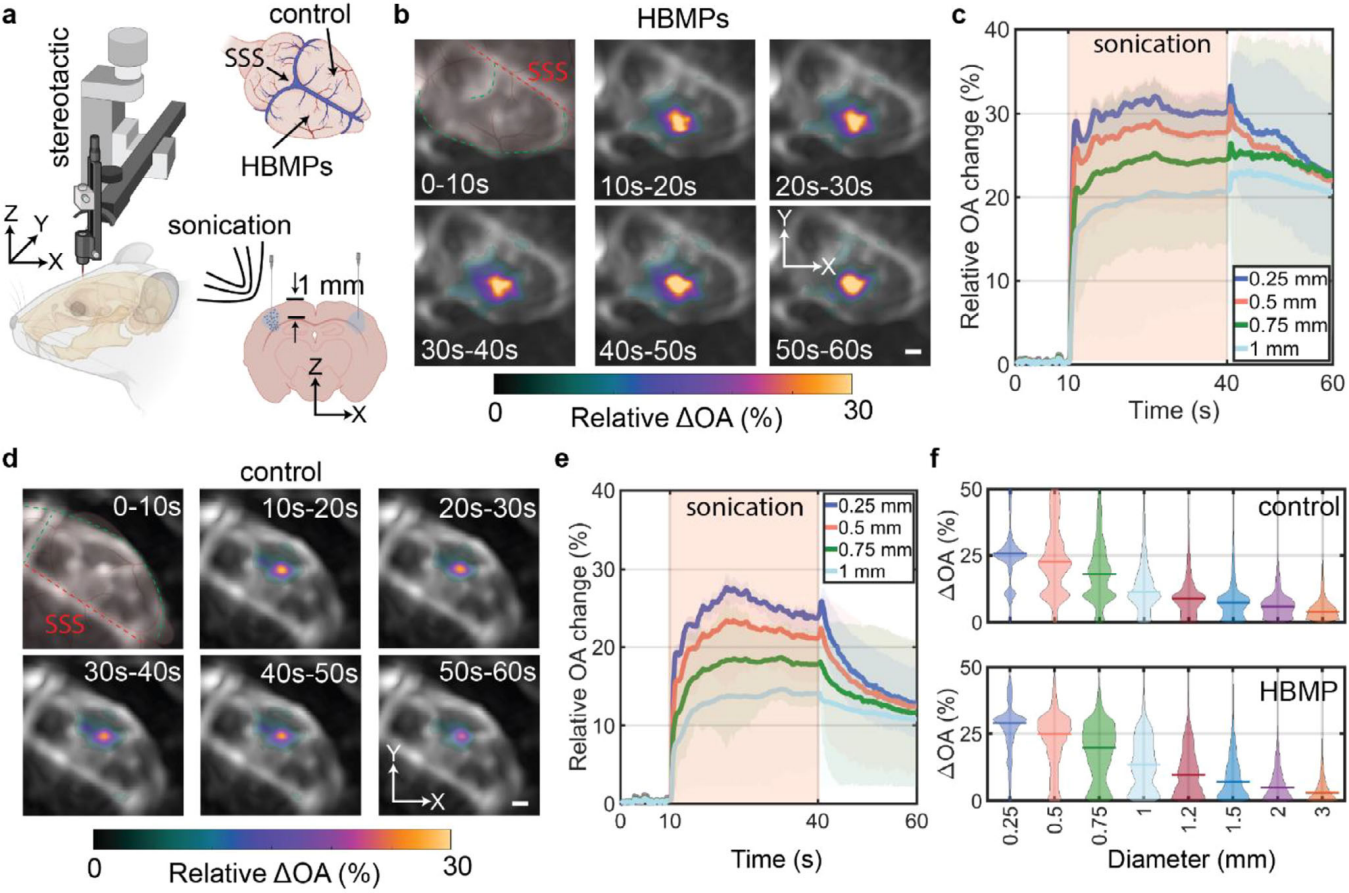
FUS‐induced lesions in ex vivo mouse brain with and without HBMPs. (a) A stereotactic apparatus was used to inject 2 µL of HBMP suspension (0.2%) and PBS‐only into the brain tissue through a pre‐drilled cranial opening. (b) Relative OA signal changes in the HBMP‐injected hemisphere across multiple time intervals. MIPs of volumetric data are shown. Scale bar: 1 mm. (c) Time‐lapse plots of the relative ΔOA_n_ measured within spherical ROIs of varying diameters, centered at the FUS focus in HBMP‐treated samples. The lines represent the mean values, and the shaded areas indicate the range from minimum to maximum within the ROI. (d) Relative OA signal changes in the PBS‐only‐injected hemisphere across multiple time intervals. Scale bar: 1 mm. (e) The corresponding time‐lapse plots measured within spherical ROIs of varying diameters for control experiments. The lines represent the mean values, and the shaded areas indicate the range from minimum to maximum. (f) Temporally averaged ΔOA_n_ for each individual point of the ROI over the full sonication period (10–40 s), plotted as a function of ROI diameter. The results show the mean and distribution of four different samples.

Freshly euthanized mice were positioned on a manual holder, and a 2‐µL particle suspension was injected into the brain using a stereotactic device through a submillimeter hole drilled in the skull (Figure 5a). Subsequently, samples were placed in the FUS‐OA setup (Figure 4a), and time‐lapse OA data were acquired. The sonication sequence remains consistent with previous protocols. Experiments were conducted using two sets of conditions: one hemisphere was exposed to both HBMPs and FUS, while the contralateral hemisphere served as a control, receiving an injection of PBS‐only followed by FUS sonication. HBMPs enhance the therapeutic efficacy of FUS primarily by optimizing localized heat deposition at targeted dosages and facilitating heat convection into regions beyond the immediate FUS focal volume (Figure 5b).

Time‐resolved sequences of the OA signal acquired from four ROIs demonstrate a rapid initial increase in OA intensity, indicative of heat generation and corresponding temperature elevation (Figure 5c). This initial rise is followed by an abrupt change, correlating with alterations in tissue optical properties, indicative of the onset of tissue coagulation (Figure 5c). Control experiments exhibit significantly smaller changes in the OA signal compared to experiments involving HBMPs (Figure 5d,e). Spatially averaged ΔOA within an ROI across four distinct tests—where ROIs are defined as spherical shells—measured 10 s after FUS, exhibits a decreasing ΔOA trend with increasing ROI size (Figure 5f). The introduction of HBMPs results in a significant increase in ΔOA compared to control conditions (Figure 5f), indicating that the particles enhance the effects of FUS and expand the affected volume (Figure 5b,d).

**FIGURE 6:**
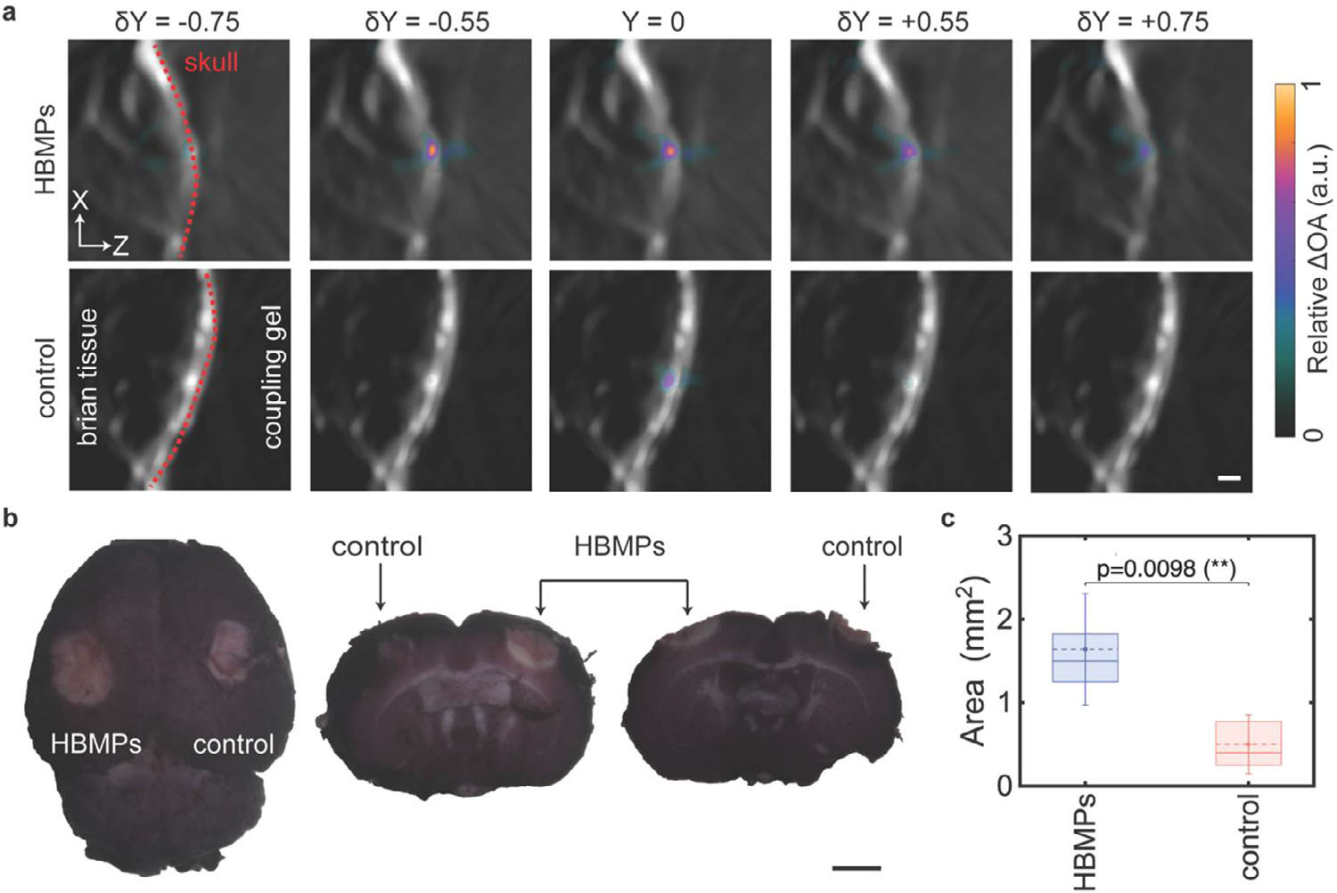
Characterization of the lesion and corresponding histological analysis. (a) Coronal OA images and relative signal changes for HBMP‐treated and control (PBS‐only) samples, acquired 10 s after the onset of sonication. Scale bar: 1 mm. (b) Histological analysis of the ablated brain tissue, bright‐stained regions corresponding to coagulated tissue in the right (control) and left (HBMP) hemispheres. Scale bar: 2 mm. (c) Mean lesion size following sonication, with standard deviation calculated across four independent experiments (n = 4). A one‐way ANOVA shows a statistically significant difference (*p* = 0.009) between lesions induced by FUS alone and FUS in the presence of HBMPs.

Coronal views (Figure 6a) indicate that the area affected in experiments with HBMPs measures approximately 1 mm^2^, which is considerably larger than that observed in control experiments. Furthermore, comparison of ΔOA along the axial direction reveals an enhanced penetration depth and broader affected regions, in contrast to control experiments, where changes are primarily confined to the surface adjacent to the skull, corresponding to the area of highest sound pressure intensity.

Subsequent histological analysis (see Materials and Methods) was conducted to assess alterations in tissue structure following sonication. After brain extraction and preparation, tissues were sectioned coronally and stained. Notably, an elliptical bright region indicative of damaged tissue was observed at the lesion site (Figure 6b; Figures S11 and S12). Histological analysis revealed that brighter regions correspond to coagulated tissue, whereas darker regions represent unaffected tissue. Coronal histological sections further corroborated the OA findings, demonstrating that the inclusion of HBMPs significantly enlarges the ablated area, particularly by increasing its depth compared to control conditions. Histology analysis indicates a threefold increase in the size of the ablated tissue with HBMPs+FUS, compared with FUS alone (Figure 6c).

## 3 Conclusion

FUS holds great promise for noninvasive treatment of neurological disorders, including brain tumors [45]. However, its efficacy is hindered by skull‐induced acoustic losses and risks of uncontrolled cavitation, which compromise safety and precision [46, 47]. Microbubble integration can enhance therapeutic effects at lower intensities, but their instability, short lifespan, and potential to cause hemorrhage or edema limit clinical utility [48]. To address these limitations, we investigated a microparticle‐mediated strategy using hollow borosilicate microparticles—HBMPs (Figure 1). These particles share key acoustic properties with microbubbles, a negative acoustic contrast factor, but provide greater mechanical stability and enable non‐cavitational, thermally mediated treatment. We characterized their acoustic behavior under FUS exposure, drawing on principles established in microbubble physics. Our findings revealed a concentration‐dependent response, with HBMPs acting either as thermal emitters or reflectors depending on the administered dose, thereby enabling tunable heat deposition and precise control of the thermal response (Figure 2). Numerical simulations showed that both the primary oscillations of individual HBMPs and secondary interparticle interactions contribute to enhanced acoustic attenuation in tissue, leading to localized temperature increases (Figure 3). In addition, we observed frequency‐dependent thermal responses consistent with resonance behavior, indicating that HBMPs can be engineered for multifunctional therapeutic applications. By tailoring the US window of parameters, we demonstrated the ability to trap HBMPs under high‐flow conditions and activate their heating function under a different regime to ablate the tissue (Figures 4 and 5). Using a waveform optimized for thermal energy deposition, we achieved a threefold increase in ablation volume in ex vivo mouse brain tissue compared with particle‐free controls after stereotactic injection of the particle suspension (Figure 6). Collectively, these findings demonstrate the potential of microparticle–based strategies as ultrasound agents, providing enhanced mechanical stability while preserving key acoustic characteristics analogous to those of microbubbles.

Despite these promising findings, several key challenges must be addressed before preclinical validation can be fully accomplished. These include evaluating long‐term biocompatibility post‐surgery, establishing particle biodegradability, optimizing particle size, and advancing real‐time, accurate temperature‐monitoring methodologies. These represent critical technical barriers that require innovations in imaging, fabrication, and sensing technologies to mitigate unintended collateral tissue damage in vivo. Our approach has demonstrated measurable long‐term biocompatibility in vivo;[49, 50, 51] however, the current particle formulation lacks biodegradability over extended periods. Furthermore, although the currently proposed intratumoral injection strategy is practical and allows precise control of particle dosing with repeated administrations over extended treatment periods, optimizing particle size for systemic circulation and intravenous delivery could facilitate deeper tissue penetration and larger ablation volumes. Achieving this would require further refinement of particle‐specific parameters, as preliminary findings indicate that the combination of FUS and HBMPs can effectively induce localized blood coagulation (Figure S13 and Section S6) and may also serve as an intravenously administered, US‐responsive contrast agent. Future investigations will therefore focus on developing biodegradable ultrasound‐responsive microparticles with tailored, tunable size distributions optimized for the selected administration route; examining selective and controlled acoustic trapping within the cerebral vasculature; and advancing spatially precise, on‐demand thermal therapies under biologically relevant conditions. Additionally, leveraging the intrinsic temperature‐monitoring capability of optoacoustic imaging in combination with thermally responsive microparticles offers the potential for real‐time assessment of tissue alterations during therapy. In this context, the particles can serve dual functions as localized heating agents and temperature‐sensitive diagnostic probes, enabling dynamic feedback on therapeutic efficacy and tissue response throughout the treatment. Overcoming these challenges will facilitate translating this approach into in vivo studies, which are currently limited by the invasive nature of therapeutic ablation procedures and associated animal welfare concerns. Nevertheless, based on the mechanisms demonstrated here and prior in vivo FUS applications [52], the clinical translation of particle‐mediated thermal therapy appears feasible, as the particles enhance and amplify FUS effects. Forthcoming studies will also investigate the effects of blood flow, tissue viscosity, and particle property variability on heating dynamics and post‐surgery immune responses, as these parameters will be critical for optimizing ultrasound settings to ensure safe and effective clinical outcomes.

Moreover, recent advancements in microrobotic therapeutics, particularly involving microbubbles responsive to acoustic fields, represent a highly promising venue due to their superior controllability, mobility, and adaptability in dynamic biological environments [53, 54, 55]. Extending the findings of this research into microrobotic systems could enable the development of acoustically actuated devices possessing multifunctional properties, including precise locomotion [56], on‐demand drug release [56], selective cellular disruption [57, 58], and fluid mixing [59], together with controlled hyperthermic activation. These advancements have the potential to substantially enhance the efficacy and safety of FUS therapies, minimizing postoperative complications and broadening their clinical applicability using US‐responsive microrobotic systems.

## 4 Experimental Section and Methods

### 4.1 Acoustic Setup and Sonication

The experimental setup depicted in Figure 2 includes single‐element transducers with central frequencies of 0.5, 1.1, 2.0, and 3.5 MHz (Sonic Concepts, Inc., Washington, USA), thermocouples (OPTOCON, 4CH, Germany), a PDMS chamber, and a water tank. The PDMS chamber features 10 mm‐thick sidewalls and a ∼150 µm‐thick bottom layer. Thermocouples are placed at three heights (2 mm, 5 mm, and 6 mm) from the bottom of the chamber, which measures 16 mm in diameter and 10 mm in depth. They are also spaced in the xy‐plane, each separated by 120 degrees to avoid interference with the US field.

In the phantom experiments, the chamber is initially filled with deionized (DI) water to obtain baseline measurements prior to FUS sonication. Subsequently, the DI water was replaced with various US gel mixtures containing HBMPs (Cospheric LLC, United States) at different weight‐to‐weight concentrations. The sonication protocol was repeated to evaluate the thermal effects specifically induced by the HBMPs. Temperature changes were normalized relative to the average baseline temperature recorded during the pre‐sonication. The FUS protocol consisted of an initial 10‐s pre‐sonication measurement period, followed by a 30‐s sonication interval, and a 20‐s post‐sonication monitoring period.

For ex vivo experiments, the FUS focal point is precisely positioned 0.5–0.8 mm below the observed surface, and this positioning is consistently applied across both abdominal and cranial studies. The sonication protocol remains consistent with that used in phantom studies (10 s–30 s–20 s). FUS and OA sequences consist of combined laser pulses and ultrasound emissions. Different emission phases of the piezoelectric elements control the position and size of the focal region. In these experiments, a single laser pulse is followed by OA acquisition and 5 MHz ultrasound emission at a 49% duty cycle, corresponding to 49 ms of ultrasound emission at a pulse repetition frequency (PRF) of 10 Hz.

### 4.2 Acoustic Contrast Factor

The acoustic contrast factor [60] can be calculated using:

(1) $$\phi =\frac{1}{3}\;\left(\frac{\frac{5\rho_{p}}{\rho_{m}}-2}{\frac{2\rho_{p}}{\rho_{m}}+1}-\frac{k_{p}}{k_{m}}\right),$$

In which, ρ, ϕ, and *k* show the density, acoustic contrast factor, and compressibility. Subscripts *p* and *m* indicate the particle and medium, respectively. The shell effect can be incorporated into the equation by including a shell‐specific density parameter to account for its influence on overall density, as ρ_*p*_ = ρ_*b*_ (1 − *a* ^3^). In this equation ρ_*b*_ and *a* are bulk density of the HBMP and core‐to‐shell radius ratio ($\frac{r_{out}}{r_{in}}$).

### 4.3 Hydrophone Measurements

The piezoelectric array was immersed in a water tank along with a hydrophone (75 µm PVDF hydrophone SN 3045, 19030 preamp, DCPS835). The power supplies were configured to operate at 3 V. The focal point was localized using a manual stage, orienting the hydrophone toward the central cavity of the array. During each experiment, a 2 mm by 2 mm area was scanned with 100 µm steps, with one primary axis fixed. The collected data were then transferred to a computer for subsequent post‐processing and pressure estimation.

### 4.4 Optoacoustic Data Collection and Image Reconstruction

The integrated FUS and OA imaging system includes a Q‐switched Nd:YAG‐pumped optical parametric oscillator laser (SpitLight, Innolas Laser GmbH, Krailing, Germany). Laser pulses at 780 nm wavelengths and 10 ns duration are emitted at a repetition rate of 10 Hz. A custom‐made spherical matrix array transducer (Imasonic SAS, France), consisting of 512 trapezoidal elements, is used to acquire the generated OA signals following laser pulsation and to transmit FUS waves. The array's spherical surface has a radius of 40 mm, and provides an angular aperture of 149°. Each array element has an elementary mean area of 12.56 mm^2^ and a central frequency of 7 MHz with >50% bandwidth.

For optimal ultrasound coupling between the transducers and the tissue, the array is positioned inside a small 3D printed water tank filled with degassed water. The tank contains a 6‐cm opening at the bottom surface, enabling both ultrasound and light transmission. This opening is sealed with a thin, transparent plastic membrane to ensure unobstructed ultrasound transmission, while ultrasound gel is applied between the membrane and the tissue to enhance coupling.

The acquired OA signals are digitized simultaneously by a custom‐made multichannel parallel data acquisition unit (DAQ, Falkenstein Mikrosysteme GmbH, Taufkirchen, Germany) at a rate of 40 megasamples per second. Subsequently, the data are transferred via a 1‐Gbps Ethernet connection and stored on a PC for further processing and reconstruction. Acquisition routines are implemented in MATLAB (R2024a, MathWorks Inc., USA) using custom‐coded scripts. The time‐resolved OA signals are first bandpass filtered between 1 and 5 MHz and deconvolved using the impulse responses of the array's sensing elements [61]. Image reconstruction is performed using a graphics processing unit implementation of a back‐projection algorithm [62].

### 4.5 Data and Statistical Analysis

The relative change in the OA signal, denoted as ΔOA_n_, is calculated using the following Equation (2):

(2) $$\Delta OA_{n}=\left(\Delta OA_{i}-OA_{\mathrm{io}}\right)/OA_{i0},$$

where *i* represents the pixel number, OA_0_ showing the mean pre‐sonication value. The ΔOA values for each individual ROI were averaged using a threshold approach, considering only those values exceeding 10% of the maximum ΔOA_n_ at each time step.

Spatial maximum intensity projection (spatial‐MIP) represents the highest intensity observed across parallel imaging planes within a defined distance range. Temporal maximum intensity projection (temporal‐MIP) depicts the highest intensity recorded for each pixel throughout the specified observation period. Images illustrating ΔOA_n_ are overlaid onto the mean OA signal obtained before sonication. In Figure 4B, relative OA signal changes are capped at a maximum normalization value of 150%.

ROIs are defined as spherical shells occupying the volume between the boundary of the previous ROI and that of the current ROI. For instance, an ROI labeled as “1 mm” refers to a spherical shell with an outer diameter of 1 mm and an inner diameter of 0.75 mm.

Statistical analysis was performed in MATLAB (MathWorks, Natick, MA) using one‐way analysis of variance (ANOVA). Raw data were pre‐processed by normalizing the signals (i.e., to their respective baseline or reference values) and time‐averaging using a moving mean filter (*movmean*). For each experiment, separate ANOVAs were conducted to compare each individual group with a designated reference group (two‐level factor: test group vs. pooled reference), using MATLAB's *anova1* function without graphical output, and the resulting two‐sided *p*‐values were reported. Because each analysis involved only two levels, no additional post‐hoc multiple‐comparison test or alpha adjustment was applied. ANOVA was used under the standard assumptions of approximate normality and homogeneity of variances. Data are presented as mean ± SD, and sample sizes (n) for each statistical analysis are reported in the corresponding figure legends and in the designated section of the manuscript. Statistical significance was defined as *p* < 0.05.

### 4.6 Numerical Model

A 2D numerical analysis was performed using COMSOL Multiphysics 6.2, utilizing the Pressure Acoustics and Bioheat Transfer modules for frequency‐ and time‐domain analyses, respectively. The Pressure Acoustics module was used to simulate the stationary acoustic fields within the tissue and water domains. The Bioheat Transfer module was subsequently used to model heat transfer dynamics within biological tissues, based on the pressure distribution.

The geometry consists of a cup‐shaped acoustic transducer array with a 65 mm radius, filled with water and featuring a central cavity with a 15 mm radius. The array is directly adjacent to a tissue sample measuring 130 mm by 65 mm, with no intervening gap. To accurately represent the influence of HBMPs within the tissue, a Narrow Region acoustic model is implemented in a defined ROI with a radius of 5 mm. The HBMPs are modeled as spherical particles, each 12 µm in diameter, arranged in a rectangular array with 20 µm spacing. Material properties assigned to these particles include the effective density (ρ  =  400 *kg*/*m* ^3^), speed of sound (*c*  =  1250 *m*/*s*), thermal conductivity (0.2 *W*/*m*.*K*), and heat capacity (*C_p_* =  830 *J*/*kg*.*K*) treated as a unified geometry. The particle array spans 200 particles along the width (X) and has a varying number of rows along the axial dimension (Z), centrally positioned within the tissue sample. The tissue is modeled as an effective density of ρ  =  1044 *kg*/*m* ^3^, speed of sound equal to *c*  =  1568 *m*/*s*, thermal conductivity as 0.59 *W*/*m*.*K*, heat capacity as *C_p_* =  3710 *J*/*kg*.*K*, dynamic viscosity of μ  =  5 *Pa*.*s*, and a ratio of specific heats of γ  =  1.15 with an initial uniform temperature of 37°C without blood perfusion.

### 4.7 Animal Preparation and Integration in the Setup

This study was performed in accordance with the Swiss Federal Act on Animal Protection. A total of 8 freshly euthanized C57BL/6 mice (3–8 weeks old) were used in this study. The mice were transferred to a stereotactic frame (Model 68801, RWD Life Science, China). Next, an incision was made in the scalp to expose the skull. Two burr holes were carefully drilled into the skull until the dura was exposed (Bregma, 1.5–1.8 mm, lateral 2 mm) using an automatic drill (Ideal Micro Drill Surgical Drill, Harvard Apparatus, USA). A volume of 2 µL of PBS, containing 0.5–1 × 10^6^ particles, was injected into the right hemisphere using a 10 µL syringe (NanoFil 10 µL Syringe, World Precision Instrument) and 33‐gauge bevelled needle (NF33BV, World Precision Instrument). The same amount of PBS was injected into the left hemisphere as a control. The injection depth in both hemispheres was approximately 0.8 mm below the dura, as precisely controlled by the stereotactic device. When the needle was touching the dura, as monitored by a microscope (PZMIII‐BS on boom stand, World Precision Instruments, Germany), the location was set to 0 as the common reference. The injection rate (500 nL/min) was controlled by an infusion pump to avoid tissue damage with a fast injection. After injection, the needle was left in place for approximately 2 min before being slowly withdrawn. The craniotomies were then labeled with a black marker pen to provide fiducial markers in the OA images, accurately guiding the FUS.

### 4.8 Histology

After each experiment, the brains were extracted and stained with 5‐bromo‐4‐chloro‐3‐indolyl‐phosphate Nitro blue tetrazolium chloride (BCIP/NBT) in the Alkaline Phosphatase Substrate solution (B1911, Sigma‐Aldrich) to first evaluate coagulation on the brain surface. After visualization of coagulated regions, the brains were sliced to a thickness of 1 mm. Then, these slices with coagulated regions were stained with BCIP/NBT again to visualize the cortical regions below the surface. Next, brain slices were placed in tissue‐culture‐treated dishes (CLS430167‐20EA, Merck, Germany) and treated with PBS, then imaged (Zeiss Lumar V12 Stereoscope, ZEN 2.3 Pro‐Blue Edition). Wide‐field images were captured using a microscope (Zeiss, Germany) to evaluate coagulated regions. Digital images were minimally processed in ImageJ to adjust brightness and contrast for visualization purposes.

## Author Contributions

N.M., A.A., M.S., and D.R. performed conceptualization. N.M., A.A., and D.R. performed methodology. N.M., Y.C., and An.A. performed the investigation. N.M. and H.E. performed data analysis. N.M. performed visualization. A.A., M.S., and D.R. performed supervision. N.M. wrote—original draft. N.M., Y.C., H.E., A.A., M.S., and D.R. wrote—review and edited.

## Funding

US National Institutes of Health (RF1‐NS126102), Innosuisse–Swiss Innovation Agency (51767.1 IP‐LS), Swiss Cancer Research (KFS‐5234‐02‐2021), Personalized Health and Related Technologies of the ETH Domain (PHRT‐582), Max Planck Society, Max Planck ETH Center for Learning Systems, ETH AI Center

## Conflicts of Interest

The authors declare no conflict of interest.

## Supporting information




**Supporting File 1**: advs73449‐sup‐0001‐SuppMat.docx.

**Supporting File 2**: advs73449‐sup‐0002‐MovieS1.mp4.

**Supporting File 3**: advs73449‐sup‐0003‐MovieS2.mp4.

**Supporting File 4**: advs73449‐sup‐0004‐MovieS3.mp4.

## Data Availability

The data that support the findings of this study are available in the supplementary material of this article.
