# Supplementary material for: Rigid Hollow Microparticles for Enhanced Focused Ultrasound Treatment Under Optoacoustic Guidance
Source: Adv Sci (Weinh). 2025 Dec 22;13(13):e12337. doi: 10.1002/advs.202512337 (PMC12955934; doi:10.1002/advs.202512337)
Supplement: Supplementary file 1 — Supporting File 1: advs73449‐sup‐0001‐SuppMat.docx. [file ADVS-13-e12337-s004.docx]

Supporting Information

**Rigid hollow microparticles for enhanced focused ultrasound treatment under optoacoustic guidance**

*Nima Mahkam, Yi Chen, Héctor Estrada, Ananya* *Amitabh, Amirreza Aghakhani,^*^ Metin Sitti,^*^ Daniel Razansky^*^*

*Corresponding author. Email: [amirreza.aghakhani@bio.uni-stuttgart.de](mailto:amirreza.aghakhani@bio.uni-stuttgart.de) (AA), [sitti@is.mpg.de](mailto:sitti@is.mpg.de) (M.S), [daniel.razansky@uzh.ch](mailto:daniel.razansky@uzh.ch) (D.R).

**This PDF file includes:**

Supplementary Text S1-S6

Figures. S1 to S13

Movies S1 to S3

References (1 to 9)

**Other Supplementary Materials for this manuscript include the following:**

Movies S1 to S3

Supplementary Text

**Section S1—Acoustic contrast factor.** The ACF can theoretically be computed using the following equations:^[1]^

| $\Phi(\kappa, \rhõ) = (\frac{1}{3})[(5\rhõ - 2)/(2\rhõ + 1) - \kappã]$, | (1) |
| --- | --- |
| $\rhõ = (\rho_{p})/(\rho_{f})$, |  |
| $\kappã = (k_{p})/(k_{f})$, |  |

the parameters are the density ratio $\rhõ$, compressibility ratio 𝜅̃. The ACF was measured by comparing particle velocity deviations after sonication with their pre-sonication values along the direction of sound propagation. The acoustic radiation force exerted on the particles and acoustic energy density are quantified using:

| $F_{rad} = 4\pi\Phi k\alpha^{3} E_{\mathrm{ac}} sin(2kz)$, | (2) |
| --- | --- |
| $E_{ac} = (p_{a}^{2}) / (4\rho_{f} c_{f}^{2}).$ |  |

Here, 𝑘 is the wave number (𝑘 = 2𝜋𝜆), 𝑎 shows the particle radius, 𝑧 describes the distance from the initial position of the particle to its final aligned position, $c_{f}$ is the speed of sound and $P_{a}$is the pressure. Comparing the forces acting on a single particle relative to the flow direction facilitates calculation of the ACF. These forces include flow-induced drag and acoustic radiation forces, which can be expressed by:

| $F_{rad}=ma+F_{drag.}$ | (3) |
| --- | --- |

The particle acceleration $a$ can be experimentally obtained from trajectory tracking, which is automatically computed by the Tracker tool using a finite difference algorithm.^[2]^ The particle mass is calculated from its known mean density and volume. The drag force is determined using the Stokes drag equation,^[3]^ where $\mu_{f}$ represents the dynamic viscosity of the fluid:

| $F_{drag}=6\pi\mu_{f}v_{x}a$ | (4) |
| --- | --- |

**Section S2—Standing wave setup.** A standing wave-based setup (Figure S2) was used to characterize the response of microparticles under varying sound wave frequencies. The trajectories of particles (Figure S4A) exposed to a 2 MHz standing wave at 0.5 KPa (Figure S3) and the corresponding velocity changes were compared with control conditions (particles in the same phantom under flow without ultrasound). Upon ultrasound sonication, particles migrated toward the antinodes due to the action of primary acoustic forces. Velocity variations before and after sonication were calculated at different sound frequencies. The velocity changes (Figure S4B) were measured in the X direction, perpendicular to the flow. These results indicate that particle migration toward the antinode occurs at frequency-dependent velocities, thereby demonstrating resonance behavior within the primary acoustic field.

**Section S3—Particle size distribution.** The HBMPs analyzed exhibited a mean size distribution of 12 μm. Particles were filtered using a 10 μm syringe filter. The results (Figure S5) show a mean size distribution of 12 μm before filtration and 5 μm after filtration, resulting in two dominant particle populations. The presence of these heterogeneous size distributions (approximately 12 μm and 5 μm) enhances radiation force interactions as well as interparticle acoustic interactions.

**Section S4—Mechanical robustness.** Theoretical investigation of indentation over a spherical shell (i.e., shell deformation), where the shell thickness (t) and deformation (δ) are considerably smaller than the radius (R), combined with the material properties of the borosilicate shell, results in an effective compressibility ($\kappã$) range of 0.1 GPa^-1^ to 0.5 GPa^-1^, depending on particle size. Using classical shell theory, plane-stress, with uniform radial expansion condition, we have:

| $k_{eff}=\frac{2Et}{3(1-\nu)R}+k_{air}$,  $\kappã=\frac{1}{k_{eff}},$ | (5) |
| --- | --- |

where *E* and *ν* represent the Young’s modulus and Poisson’s ratio of the shell, respectively. $k$ shows the effective stiffness of the HBMPs, considering both the core and shell properties.^[4]^

**Section S5—Size-dependent behavior.** For two spherical particles separated by a distance x and subjected to an ultrasound wave, their interaction can be described by the following force balance:^[5–7]^

| $F_{12}=\frac{\rho}{4\pi x^{2}}<\dot{V_{2}}\dot{V_{1}}>$, | (6) |
| --- | --- |

This expression describes the force exerted from particle 1 on particle 2, where $\rho$ is the fluid density and $V_{i}$ is the instantaneous volume of particle i. Using the Crum equation, this relation can be simplified to:

| $F_{12}=2\pi\rho\left( R_{1}R_{2}\omega\right)^{2}\frac{\vec{r_{1}}-\vec{r_{2}}}{\left\vert\vec{r_{1}}-\vec{r_{2}} \right\vert^{3}}\zeta_{1}\zeta_{2}\cos\left( \phi_{1}-\phi_{2} \right),$ | (7) |
| --- | --- |

where ω = 2πf is the angular (driving) frequency, f is the ultrasound frequency, $\vec{r_{1}}$ denotes the position vector of particle i, and $\phi$ is the oscillation phase. Each particle has an instantaneous radius of $Rᵢ + \zetaᵢ e^{-i(wt-\phi_{n})}$, where Rᵢ is the equilibrium radius and ζᵢ is the radial displacement. Assuming a single harmonically driven frequency at low amplitude:^[8]^

| $F_{12}=\frac{2\pi{\rho\omega}^{2}}{r^{2}}R_{1}^{2}R_{2}^{3}\left\vert\vec{r_{1}} \right\vert\vert\vec{r_{2}}\vert cos(\phi_{1}-\phi_{2}),$ | (8) |
| --- | --- |

**Section S6—Optoacoustic temperature estimation.** Figure S13 shows temperature estimation in phantoms including blood and particles. In these experiments, both FUS alone and FUS + HBMPs produced measurable temperature increases, with the presence of the particles further enhancing the heating effect. The FUS protocol consisted of 5 seconds of baseline monitoring, 30 seconds of sonication (from 5 to 35 seconds), and 25 seconds of post-sonication monitoring. Flow was maintained at 0.1 ml.min⁻¹ through a 1 mm inner-diameter tube. Comparisons between “blood only” and “blood + HBMPs” revealed two principal effects: (i) particles increased the overall temperature rise, and (ii) sudden signal changes—consistent with the onset of coagulation—occurred only in the presence of particles (see the green trace at t = 20 s and the orange trace at t = 32 s), and were absent in the blood-only control. The results presented in Figure S13 are estimated within a temperature range of 20 °C to 150 °C.

Temperature estimates were derived from the OA signal via the Grüneisen parameter (detailed below), which relates OA amplitude to temperature. In the present tests, however, the OA signal is affected not only by temperature but also by FUS-induced red blood cell (RBC) redistribution and associated flow dynamics. Accordingly, the OA-based temperature estimations here should be interpreted as relative temperature indicators rather than absolute measurements.

Temperature change estimating using Grüneisen parameter and OA can be derived using:^[9]^

| $\Gamma\left( T \right)=\Gamma_{0}+\alpha T,$  $\Delta T=\frac{\Gamma_{T}\Delta OA}{\alpha{OA}_{0}},$ | (9) |
| --- | --- |

where T is the temperature, Γ is the temperature-dependent Grüneisen parameter, with ΔOA and OA_0_ showing the relative OA change and the baseline values.

**SI References**

[1] H. Bruus, Acoustofluidics 7: the acoustic radiation force on small particles. *Lab on a Chip* **2012**, *12*, 1014.

[2] J. Riba, S. Zimmermann, et al., Technologies for Automated Single Cell Isolation. *Technologies for Automated Single Cell Isolation*, **2021**.

[3] P. B. Muller, R. Barnkob, et al., A numerical study of microparticle acoustophoresis driven by acoustic radiation forces and streaming-induced drag forces. *Lab on a Chip* **2012**, *12*, 4617.

[4] M. Hall, Bulk modulus of a fluid−filled spherical shell. *The Journal of the Acoustical Society of America* **1975**, *57*, 508.

[5] N. A. Pelekasis, A. Gaki, et al., Secondary Bjerknes forces between two bubbles and the phenomenon of acoustic streamers. *Journal of Fluid Mechanics* **2004**, *500*, 313.

[6] H. Bruus, Acoustofluidics 7: The acoustic radiation force on small particles. *Acoustofluidics 7: The acoustic radiation force on small particles*, Vol. 12, Royal Society of Chemistry, **2012**, pp. 1014–1021.

[7] M. Lanoy, C. Derec, et al., Manipulating bubbles with secondary Bjerknes forces. *Applied Physics Letters* **2015**, *107*.

[8] T. G. Leightont, A. J. Waltont, et al., Primary Bjerknes forces. *Primary Bjerknes forces*, Vol. 11, **1990**.

[9] Ç. Özsoy, B. Lafci, et al., Real-time assessment of high-intensity focused ultrasound heating and cavitation with hybrid optoacoustic ultrasound imaging. *Photoacoustics* **2023**, *31*.


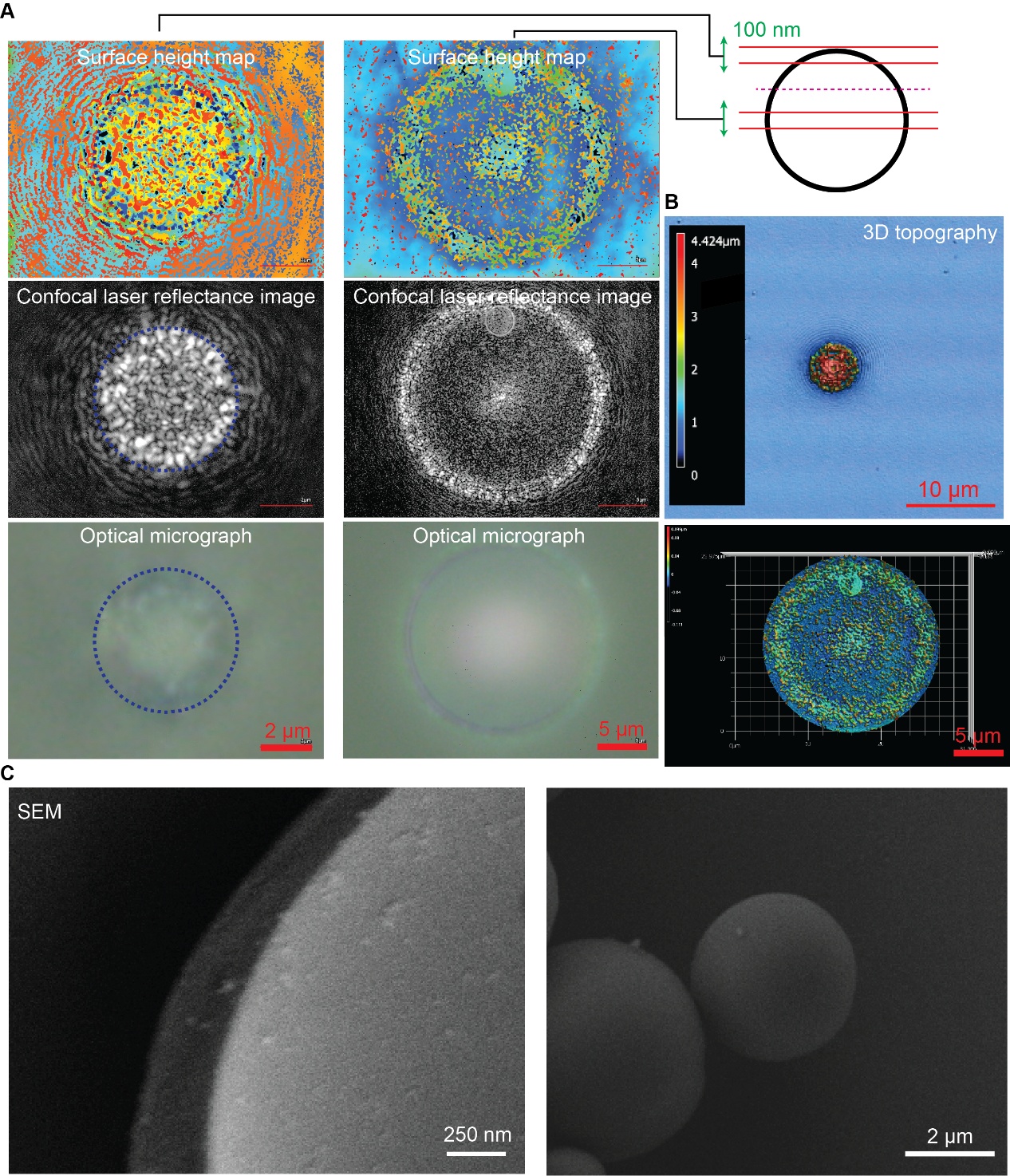


**Figure S1. Surface morphology of HBMPs.** Laser scanning confocal microscopy (VK-X Series, KEYENCE Corporation, Osaka, Japan) and scanning electron microscopy (SEM) were used to characterize shell surfaces and cross-sections. (**A-**left) Top-surface reflectance and height maps acquired at two axial planes separated by 100 nm; corresponding cross-section shown at bottom. (**A**-Right) Midline cross-section imaged, showing the laser reflectance image, optical micrograph, and height map. (**B**) 3D surface topography spanning the full particle thickness and mid cross-section. (**C**) SEM half-cut shell and fully intact particle.


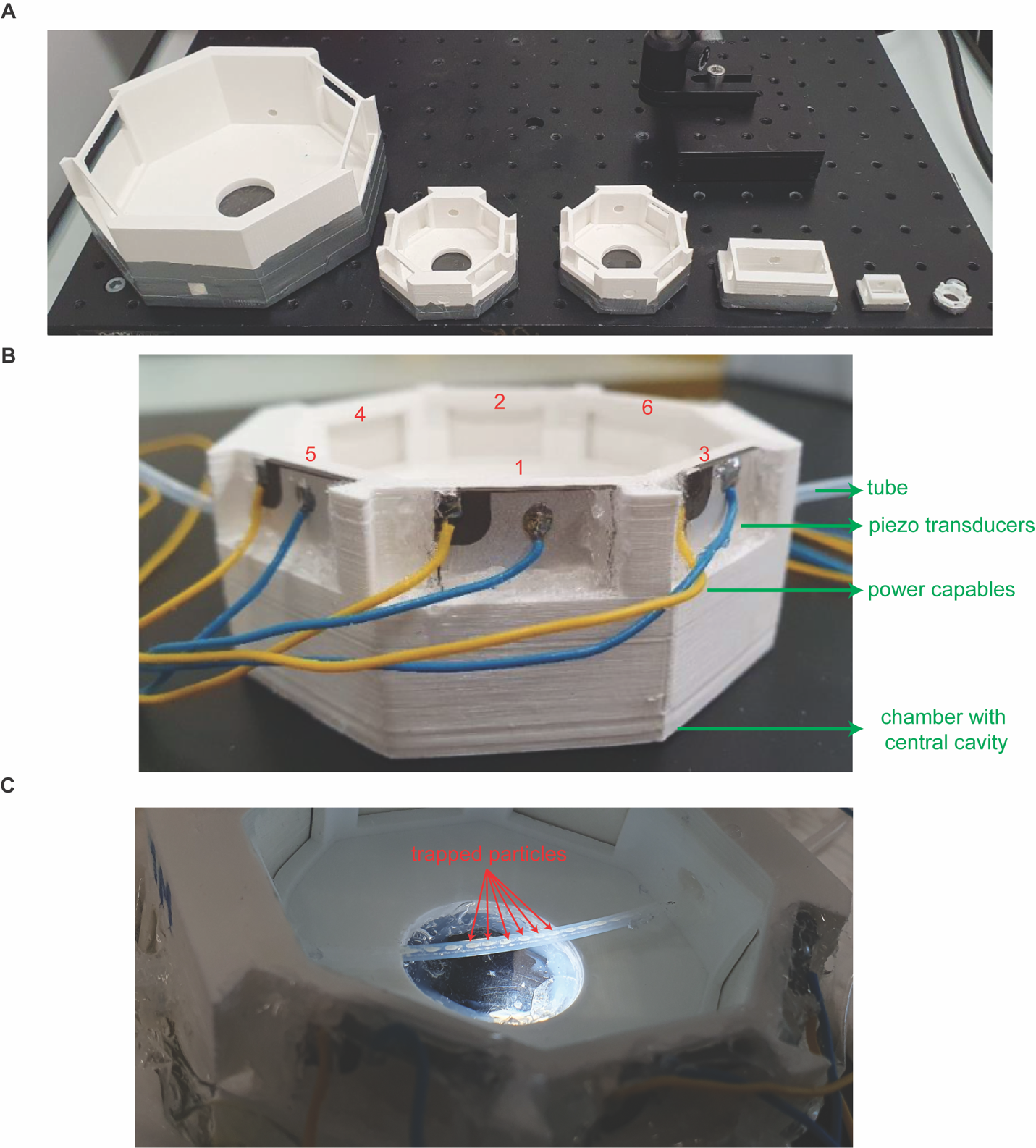
**Figure S2.** **Acoustic contrast factor measurement setup.** (**A**) The setup includes piezoelectric transducers positioned attached to the walls, a tube filled with water, and a water tank. The tube is connected to a syringe pump for continuous flow. Each chamber corresponds to 590 kHz, 1 MHz, 1.5 MHz, 2 MHz, 2.8 MHz, and 3.8 MHz. (**B**) Chamber with attached transducers. (C) Trapping of the particles post-injection at 2MHz.


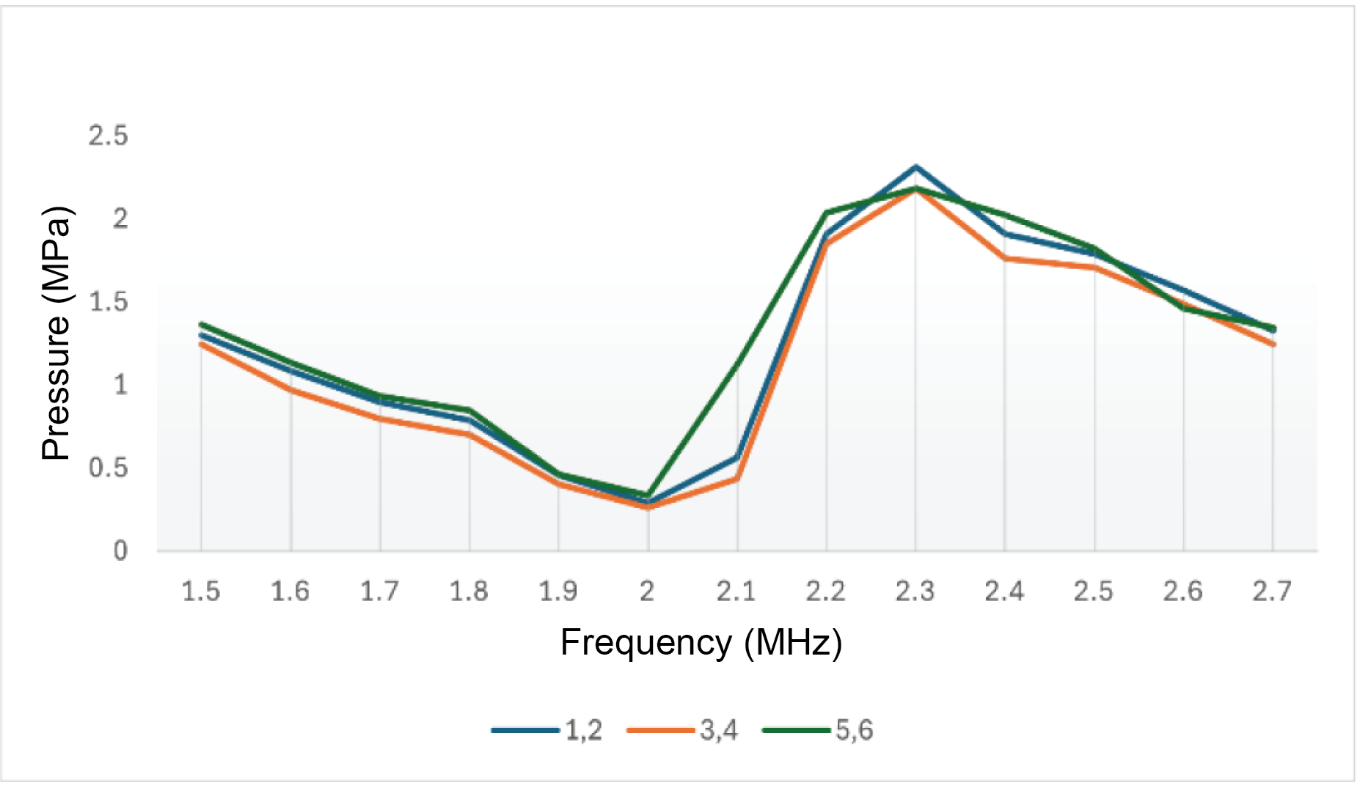


**Figure S3.** **Pressure measurements for the 2 MHz setup.** Hydrophone measurements were performed at the center of the chamber under various arrangements of piezoelectric transducer elements (transducer numbers shown in Figure S1). The results indicate a peak resonance at 2.2 MHz with a corresponding pressure of 2 MPa.


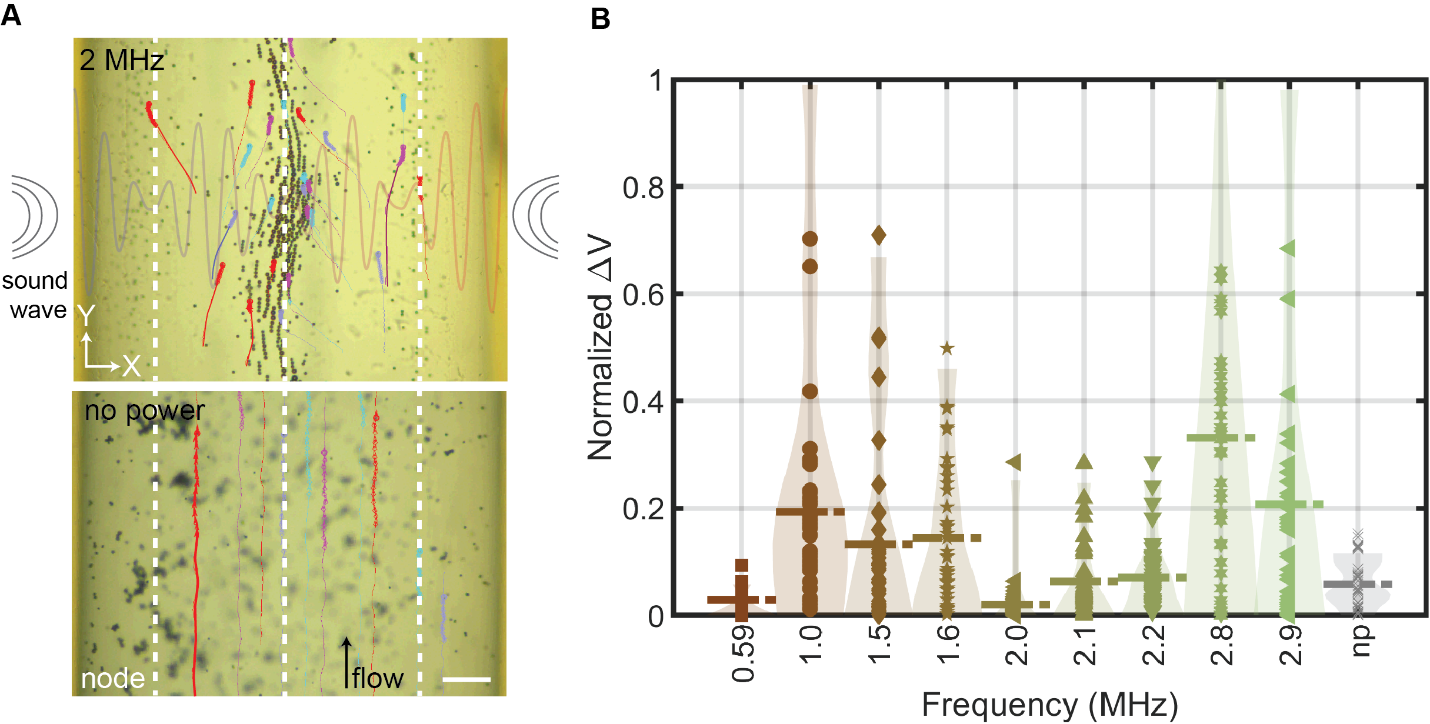


**Figure S4.** **Acoustic contrast factor measurement.** (**A**) HBMPs movement post-sonication and no-power case. (**B**) Velocity changes of HBMPs along the X-axis, perpendicular to the flow direction, at different US frequencies.


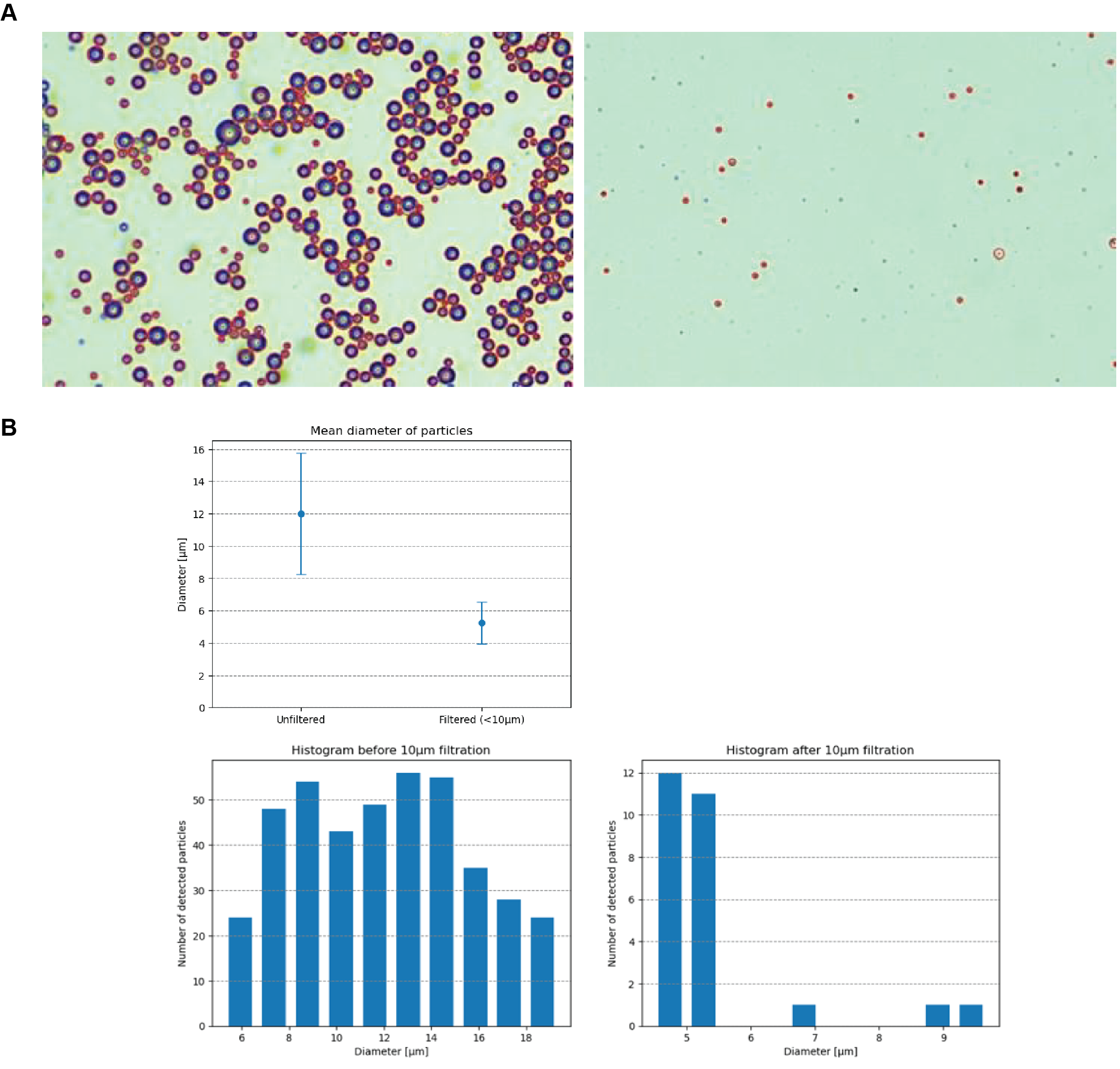


**Figure S5.** **Particle size distribution**. (**A**) Bright-field microscope images of HBMPs before and after filtration. (**B**) The size distribution of particles before and after filtration.


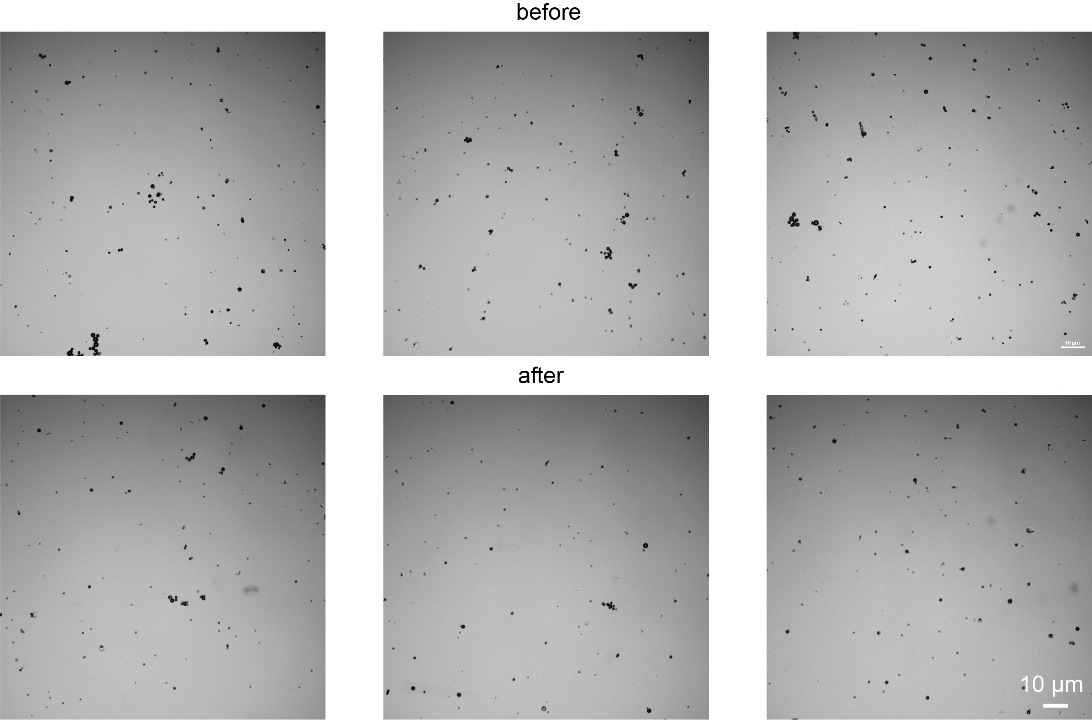


**Figure S6. Mechanical robustness of HBMPs**. Representative microscope images acquired before and after exposure to a 2-minute FUS sonication at 3 MHz, illustrating particle mechanical robustness following sonication.

**
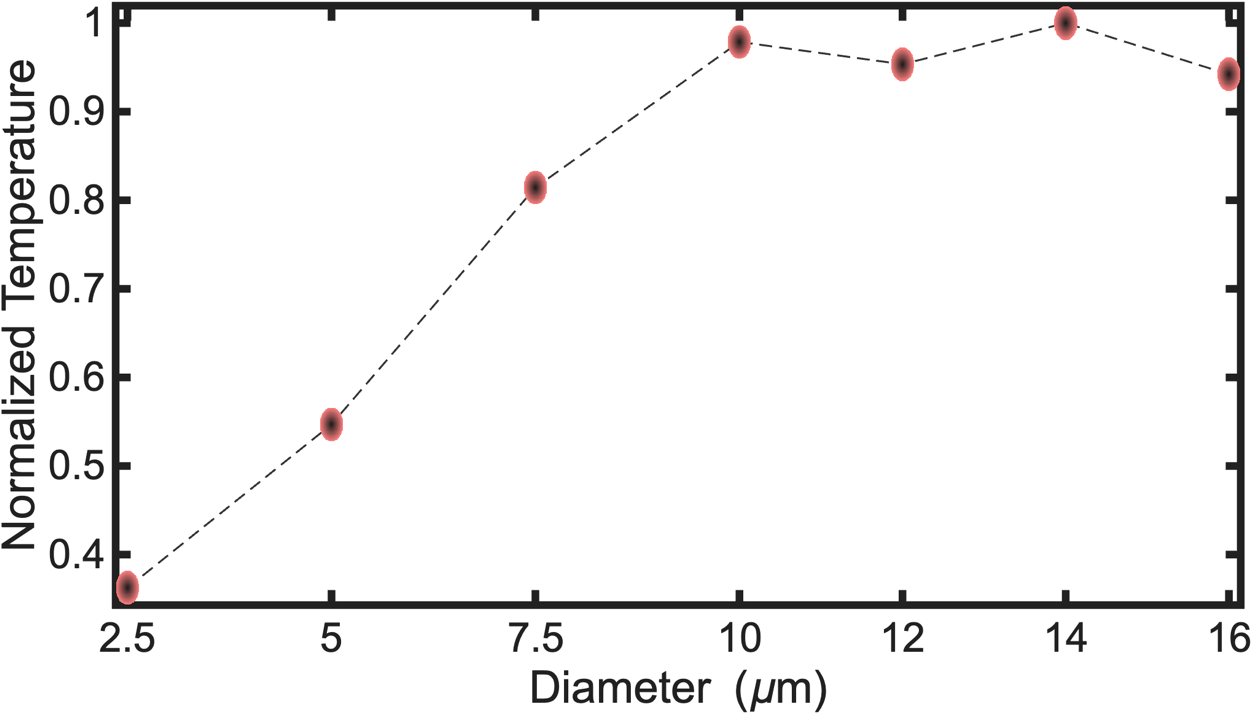
**

**Figure S7**. **Size–temperature-dependent behavior**. Numerical investigation of temperature response to changes in particle size at constant pressure and frequency (5 MHz). The number of particles (*N_r_* = 400) and their interparticle distance (20 µm) were kept constant across all numerical models.


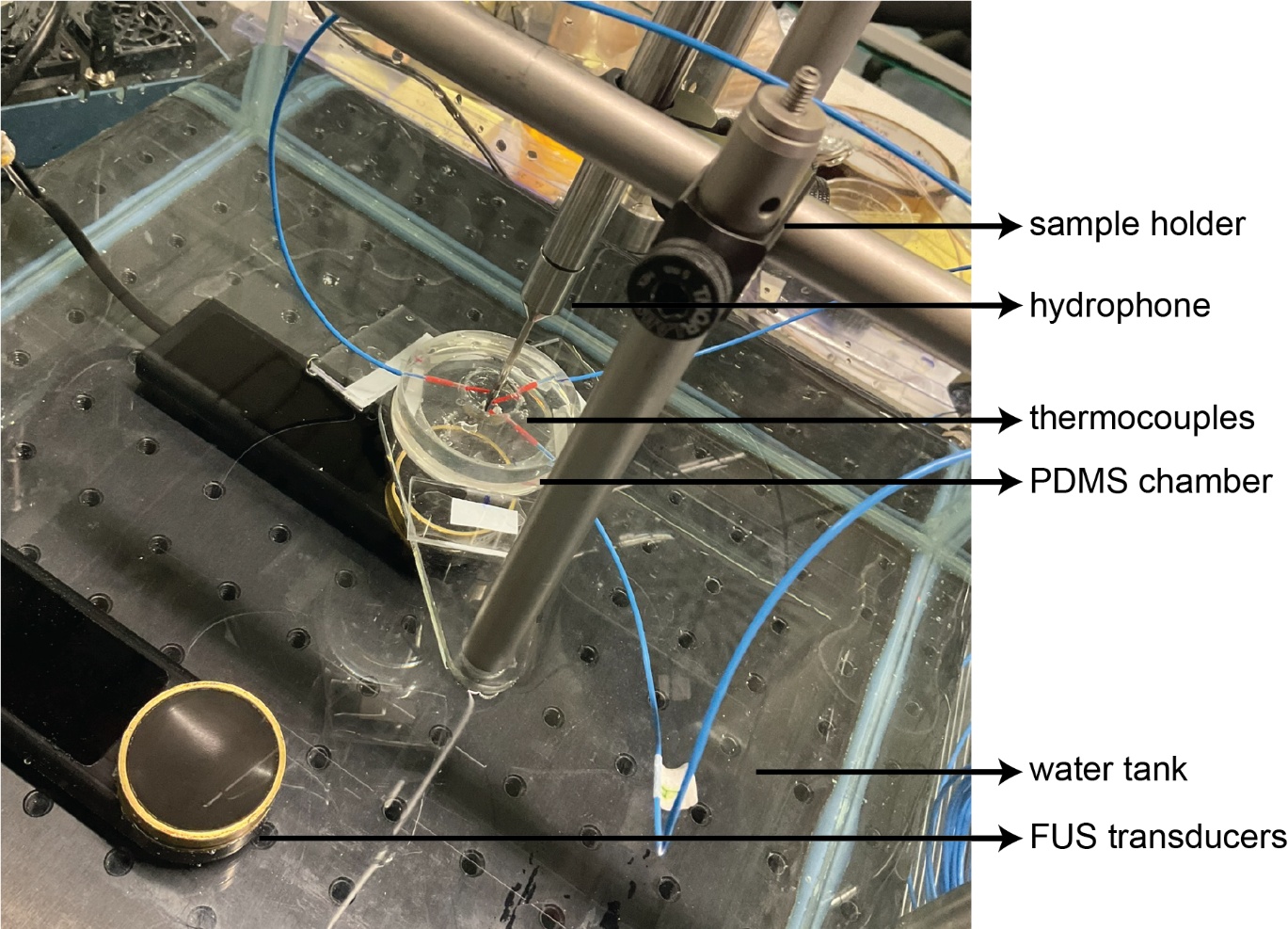


**Figure S8.** **Single-element FUS transducer setup.** The acoustic setup consists of a single-element transducer submerged in a water tank, a sample holder, a PDMS chamber featuring a central cavity with a bottom wall thickness of 100 μm, a signal generator, a high-amplitude power amplifier, a hydrophone, and optical thermocouples.


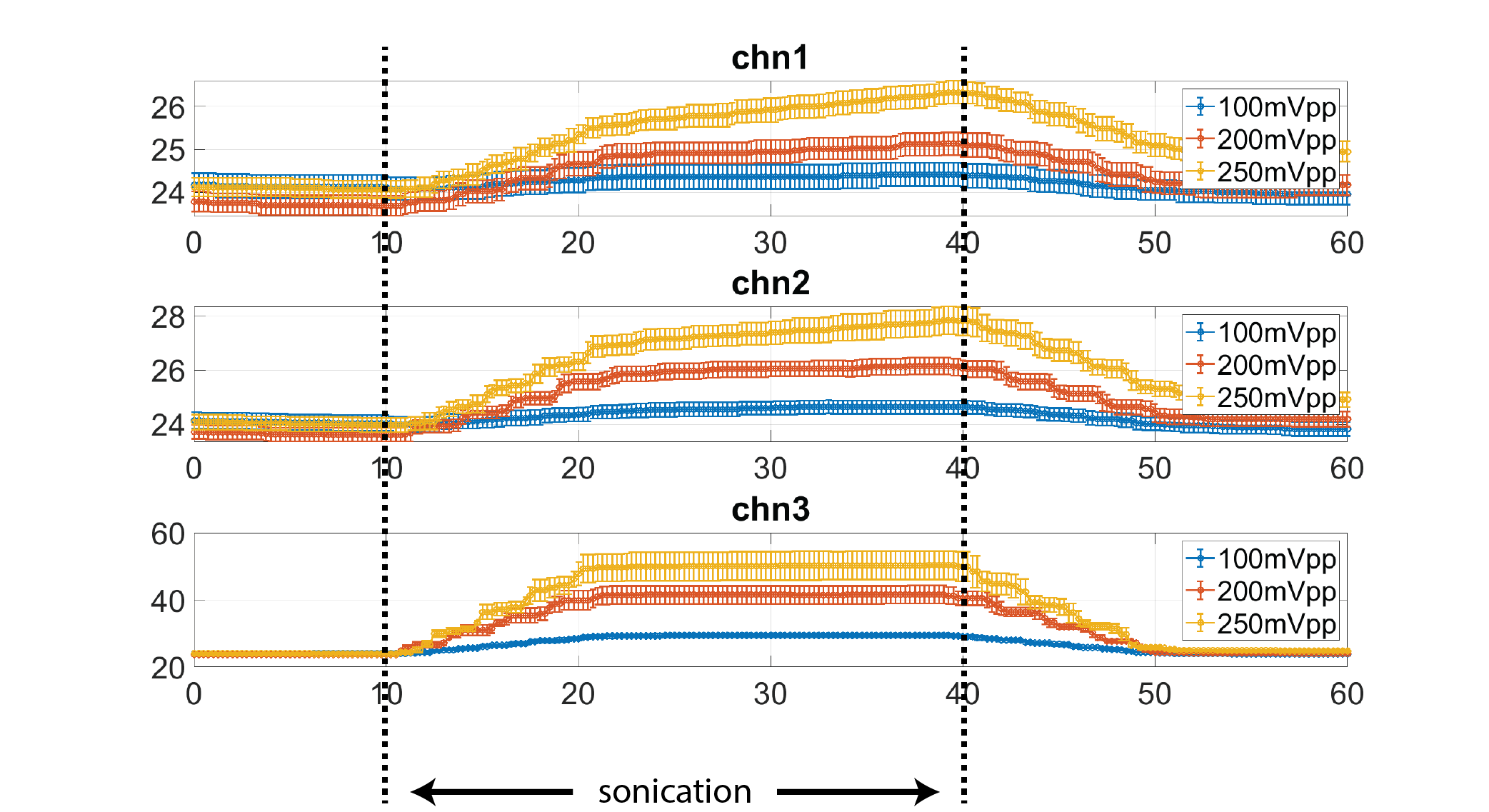


**Figure S9.** **Raw thermocouple reading of the water baseline at 2 MHz.** Show case for a temperature recordings from three thermocouples spatially distributed within the phantom demonstrate localized heat deposition during sonication and its pressure dependence.

**
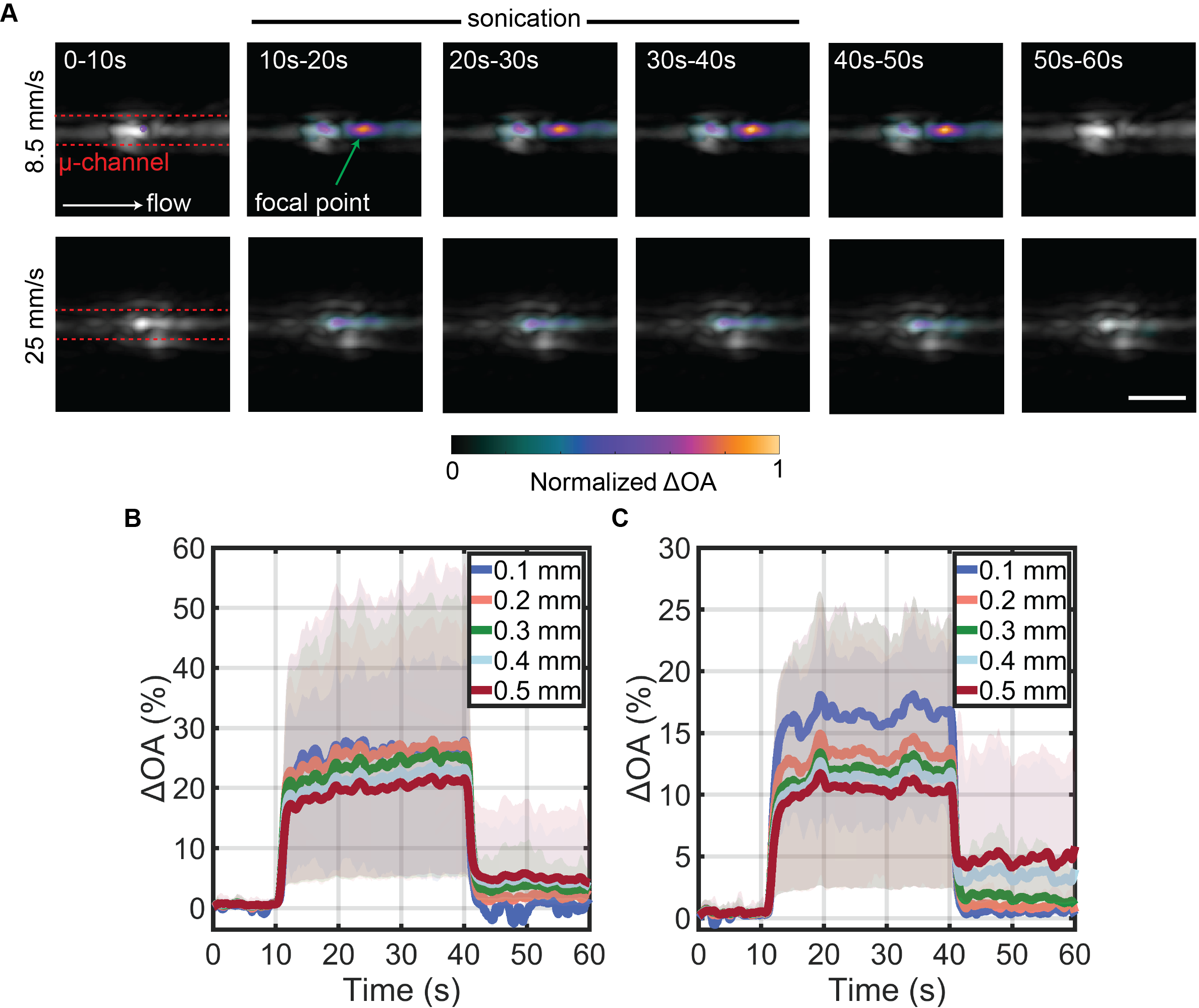
**

**Figure S10. HBMPs FUS trapping experiments. (A)** Time-lapse spatial-MIP images for flow velocities of 8.5 mm/s and 25 mm/s. The setup incorporates a syringe pump, an OA-FUS device, and a 0.5-mm tube. Sonication parameters are set to 3 MHz frequency with a 30-s duration. Scale bar: 1 mm. (**B,C**) ΔOA_n_ measured during trapping shows an increase in the OA signal at the focal point and a return to baseline once FUS is turned off, for flow speeds of 8.5 mm/s (B) and 20 mm/s (C). Average OA signal in a ROI centered on the focus point with different diameters. The particles are coated with polydopamine to enhance their optical absorption and enable OA visibility.


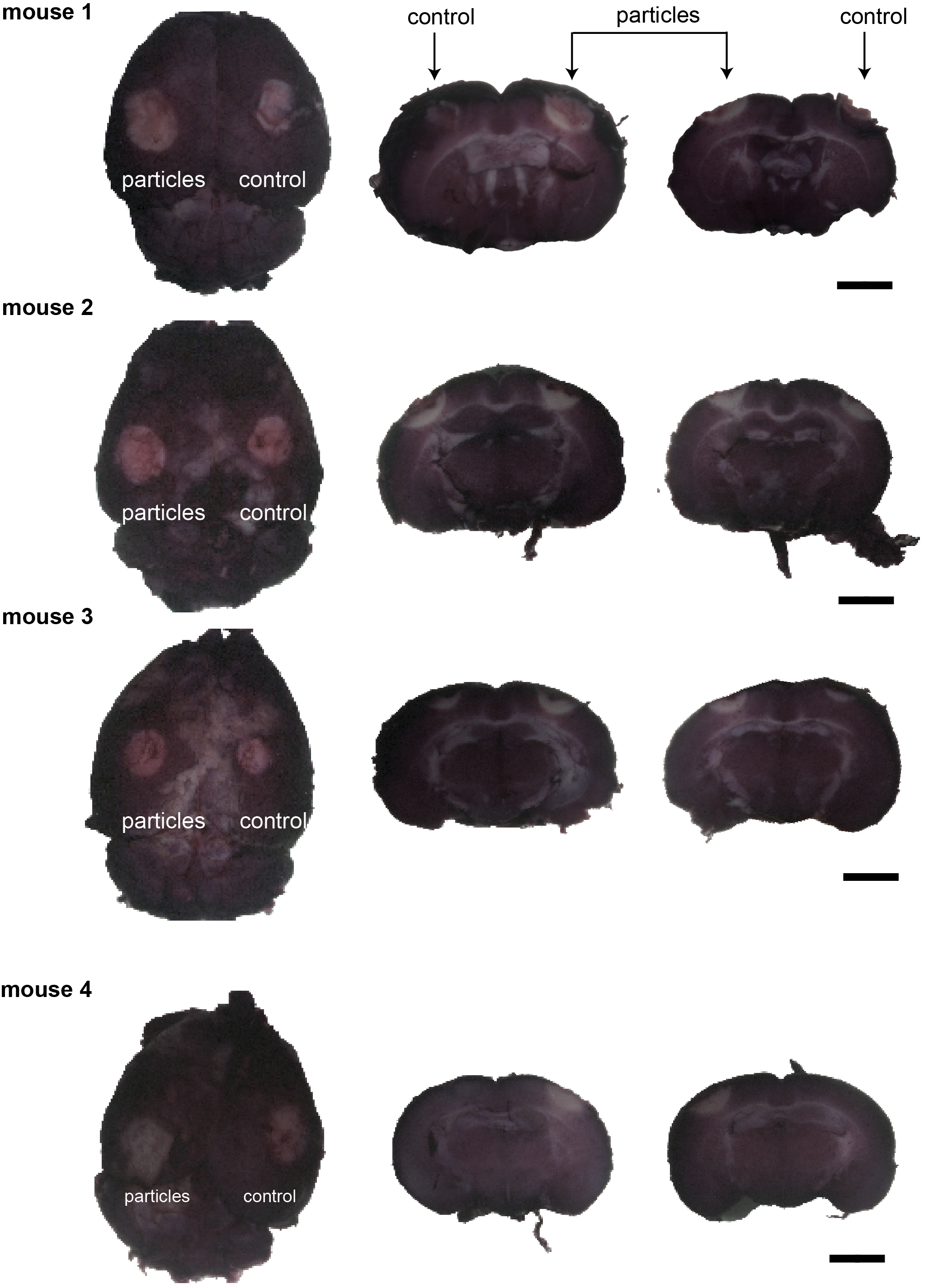


**Figure S11**. **Histology.** Stained brain samples after experiments show distinct bright and dark regions corresponding to ablated and non-ablated tissue, respectively.


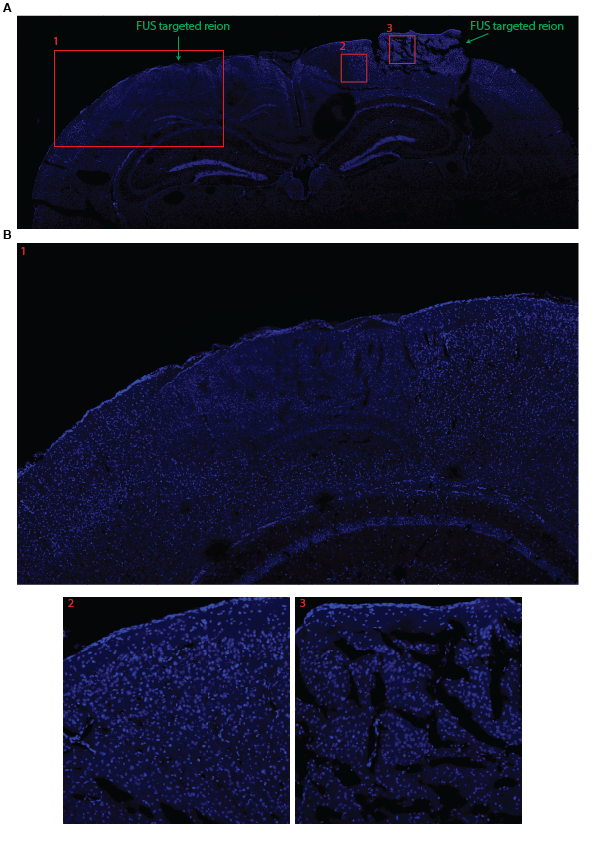


**Figure S12**. **DAPI-stained 20-µm tissue sections.** (**A**) Whole-brain DAPI-stained image showing nuclear distribution. A localized reduction in DAPI fluorescence intensity is observed in both the left and right hemispheres at FUS-targeted regions. (**B**) Higher-magnification views of the indicated regions. (1) the left hemisphere (ablated site), (2) the contralateral hemisphere adjacent to the ablation, and (3) the right hemisphere (ablated site). Sections 1 and 3 show reduced DAPI signal, irregular nuclear morphology, and areas of missing tissue, consistent with localized tissue disruption. Section 2 exhibits unaltered nuclear density, indicative of intact, non-ablated tissue.


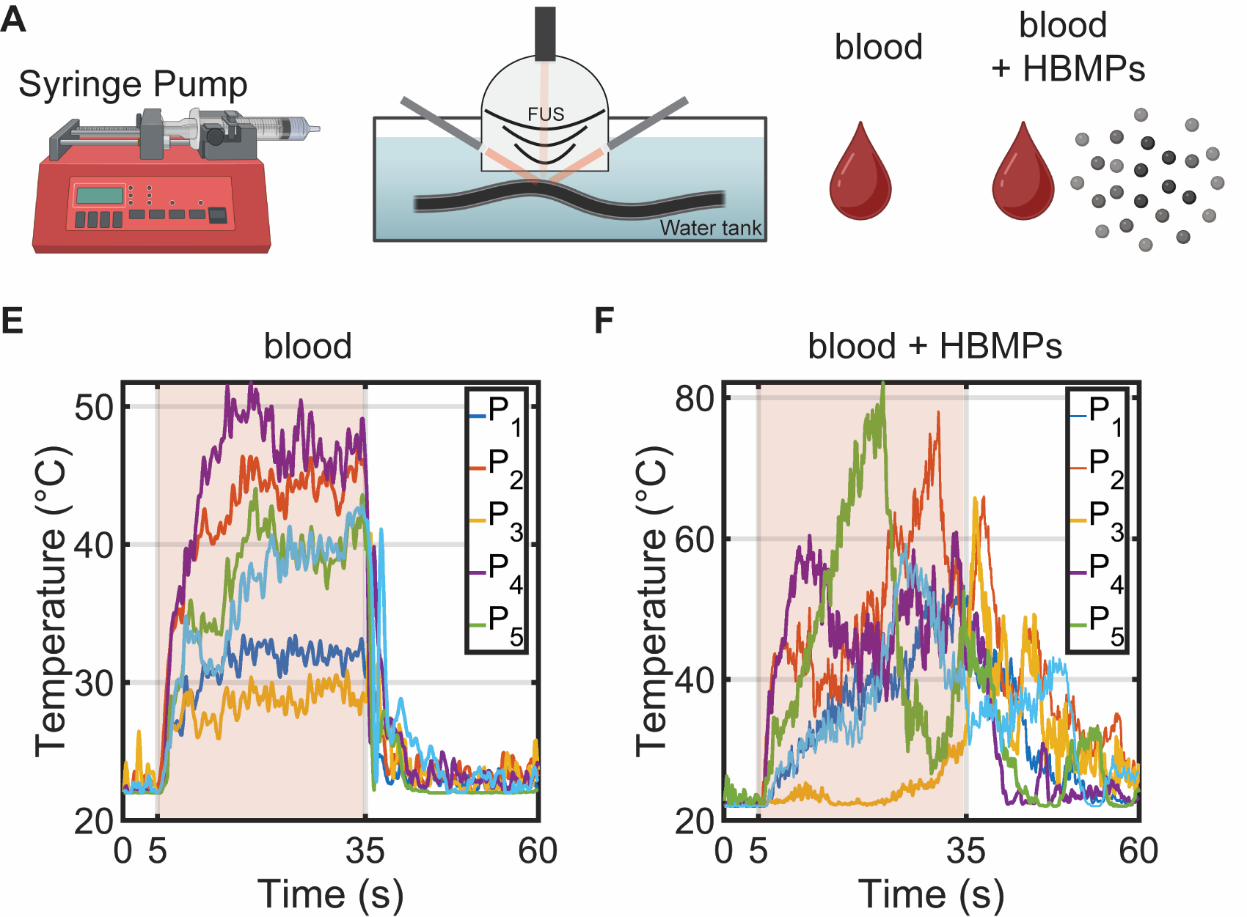


**Figure S13**. **HBMP-induced heating and flow effects**. The FUS protocol comprised 5 s of pre-sonication monitoring, 30 s of sonication, and 25 s of post-sonication monitoring. Flow is fixed at 0.1 ml·min⁻¹ through a 1 mm inner-diameter tube. The temperature was estimated using the Grüneisen parameter, assuming Γ = 0.15 at T0 = 22 °C and a temperature coefficient α = 0.004. Estimates were truncated at 150° C. The sudden trend change observed in the blood and HBMP is linked with blood coagulation. Points of interest (P_i_) are placed at multiple positions along the tube centerline, and OA values for temperature estimation were computed as the mean within spherical ROIs (radius = 250 µm) centered at each P_i_.

**Movie S1**. Physics study: Particle manipulation using a standing wave setup.

**Movie S2**. Acoustic trapping of HBMPs under Flow Conditions.

**Movie S3**. Optoacoustic tomography of brain tissue subjected to HBMPs with FUS and to FUS-only conditions.
